# Cell-based immunotherapies for infectious diseases: current evidence and translational perspectives on cytokine induced killer (CIK) cells

**DOI:** 10.1007/s00281-026-01092-x

**Published:** 2026-11-13

**Authors:** Rohulla Vaseq, Nahid Mahleqa, Arefa Mohammadi, Marc P. Hübner, Hans Weiher, Ingo G.H. Schmidt-Wolf

**Affiliations:** 1 Department of Integrated Oncology, Center for Integrated Oncology (CIO), University of Bonn, University Hospital Bonn, 53127 Bonn, Germany; 2 Public Health Faculty, Kabul University of Medical Sciences, Kabul, Afghanistan; 3 Institute for Medical Microbiology, Immunology and Parasitology, University of Bonn, University Hospital Bonn, 53127 Bonn, Germany; 4 German Center for Infection Research (DZIF), partner site Bonn-Cologne, Bonn, Germany; 5 Department of Applied Natural Sciences, Bonn-Rhein-Sieg University of Applied Sciences, Rheinbach, Germany

**Keywords:** Infection, Pathogen, CTLs, γδT cells, NK cells, CIK cells, Immunotherapy, Cell therapy

## Abstract

The persistent global burden of infectious diseases, compounded by environmental challenges, emerging pathogens, and antimicrobial resistance, underscores the urgent need for novel therapeutic strategies beyond conventional antimicrobial agents. Cell based immunotherapies, which harness the body’s immune system to combat pathogens, have gained increasing attention for their potential to provide targeted, durable, and adaptive responses. Recently, we demonstrated that clinically licensed cytokine-induced killer (CIK) cells, used in cancer treatment, significantly reduced *Plasmodium*-infected erythrocytes in vitro. This review focuses on the emerging role of CIK cells in infectious disease therapy, positioning them alongside other prominent immune effector cells such as CTLs, γδT cells, and NK cells. We explore the biology, recognition mechanisms, their potential feature, and cytotoxic functions of these cell populations, distinguishing direct findings in infection-specific CIK studies from mechanisms characterized in oncology or other cytotoxic lymphocytes. While CTLs, γδT cells, and NK cells have been extensively studied for their roles in early and adaptive immune responses, CIK cells traditionally investigated in oncology, are now being recognized for their potential in antiviral and antimicrobial immunity. Alongside with CTLs, γδT cells, and NK cells, we detail CIK cell phenotypes, activation strategies, effector functions, and the limited but growing body of evidence supporting their application in some viral infections. The available findings support further investigation of CIK cells as a candidate approach in selected infectious settings, while their broader antimicrobial applicability, comparative efficacy, target specificity, safety, and clinical benefit require further evaluation in these settings.

## Introduction

Infectious diseases, caused by a diverse array of pathogenic microorganisms including bacteria, viruses, fungi, and parasites, or their products, remain a persistent global health challenge. Throughout human history, these pathogens, transmitted via direct contact, vectors, fomites, or environmental reservoirs, have been the dominant drivers of morbidity and mortality. The 20th century marked a golden age of public health, witnessing dramatic reductions in disease burden through the advent of sanitation, mass vaccination programs, and the discovery of antimicrobials. However, these historic gains are now endangered by a constellation of converging biological, environmental and political challenges that threaten to usher in a post-antibiotic era.

Current therapeutic landscapes are increasingly fragile. The efficacy of conventional treatments is being systematically undermined by the rapid emerging and re-emerging pathogens due to evolution of antibiotic resistance, the emergence of novel viral mutations, and the rise of antifungal resistance particularly to azoles, alongside antigenic variation in parasites [1]. High-priority pathogens such as *Mycobacterium tuberculosis*, HIV, hepatitis B and C viruses, *Plasmodium* spp. and hemorrhagic fever viruses like *Ebola* and *Zika* continue to elude complete elimination. Compounding these individual pathogen threats is the accelerating global crisis of antimicrobial resistance (AMR), which the World Health Organization (WHO) has categorized as one of the top 10 global health threats facing humanity. In 2019 alone, bacterial AMR was directly associated with an estimated 4.95 million deaths worldwide, a figure projected to rise precipitously without intervention [2, 3]. These biological pressures are exacerbated by anthropogenic factors: lifestyle shifts, rapid urbanization, climate change, mass migration, and global air travel facilitate the zoonotic spillover and rapid dissemination of infections, reaffirming their status as a dynamic and evolving global threat. Immunosuppression further compounds the burden of infectious diseases, particularly in patients receiving immunomodulatory therapies, with vulnerability to infection influenced by the therapeutic mediator, patient characteristics, and concomitant immunosuppressive treatment [1, 4, 5]. Furthermore, the COVID-19 pandemic exposed the systemic vulnerabilities of modern healthcare, highlighting the critical need for adaptable, broad-spectrum strategies to combat “Disease X”, a placeholder for future, unknown pathogenic threats. Consequently, the development of alternative therapeutic modalities that transcend traditional small-molecule drugs is no longer optional but imperative.

**Fig. 1:**
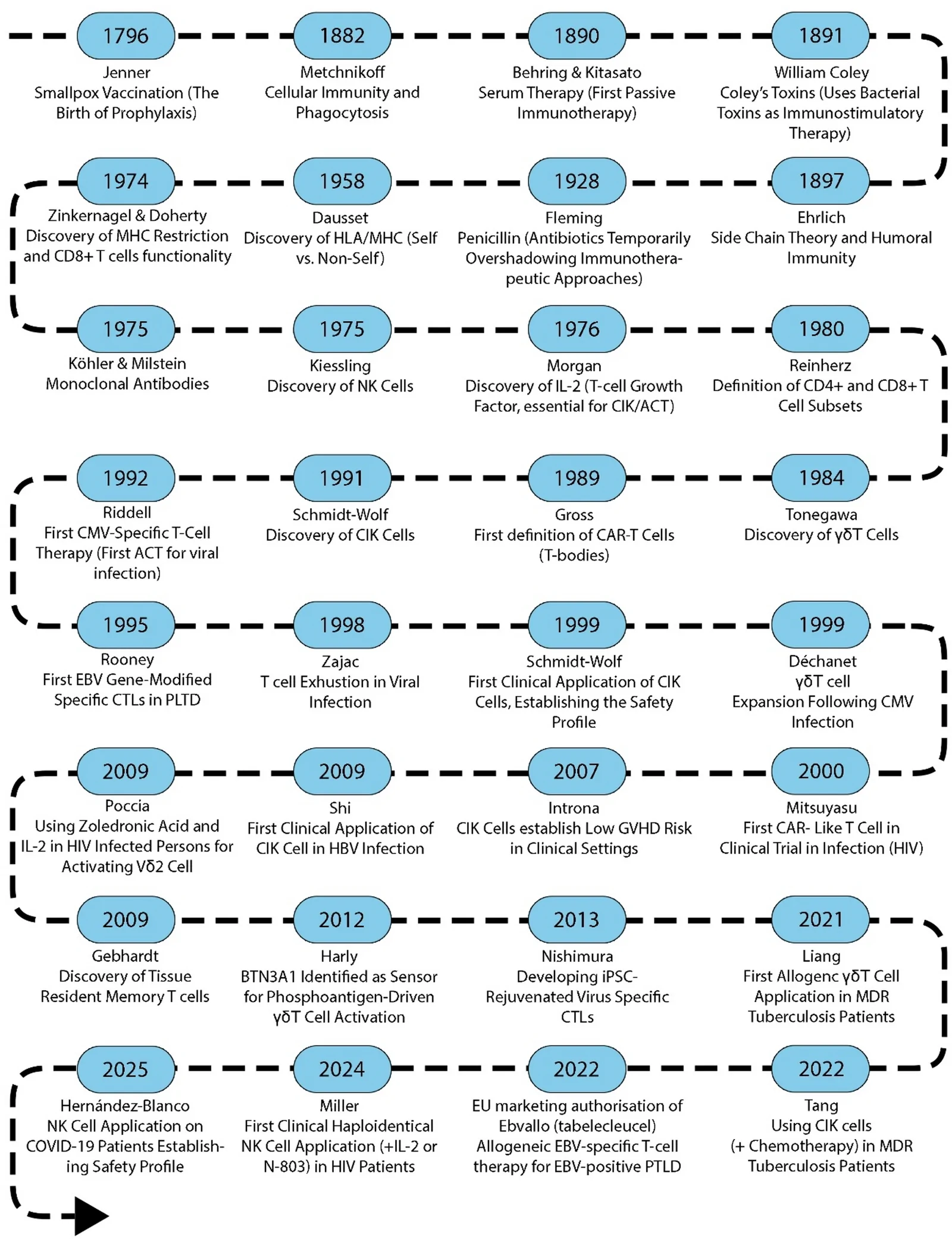
From prophylaxis to precision cell therapy. The timeline tracks the evolution of immunological strategies against infection diseases, from foundation and system defining to cellular era and marketing approval. (References otherwise not mentioned in the text [13–15])

The concept of harnessing the host’s own immune system to combat disease is not new; it is deeply rooted in medical history (Fig. 1). Edward Jenner’s seminal work on smallpox immunization in 1796 laid the foundation for prophylaxis, a paradigm that dominated immunology for centuries [6]. The therapeutic -rather than prophylactic- potential of the immune system began to crystallize at the turn of the 20th century. The pioneering discoveries of Elie Metchnikoff (cellular immunity/phagocytosis) and Emil von Behring (serum therapy) formally recognized the dual arms of immunity [7–9]. Crucially, the timeline of immunotherapy highlights the visionary work of William Coley in 1891, who observed that Coley’s toxins, the mixture of bacterial products, could induce tumor regression by stimulating the immune system. This was an early, critical hint that infectious agents and immune activation were deeply intertwined [10]. Immunology concepts were further refined by Paul Ehrlich’s side-chain theory and the study of humoral immunity [9, 11, 12].

The latter half of the 20th century witnessed a technological revolution in immunology. The definition of distinct lymphocyte subsets (T cells, B cells, NK cells, γδT cells), the advent of monoclonal antibody technology by Köhler and Milstein (1975), and the discovery of MHC restriction by Zinkernagel and Doherty (1974) provided the granular understanding necessary for precision engineering such as CAR-like T cells [16–24]. These breakthroughs transformed immunology from a descriptive science into a manipulative one, paving the way for adoptive cell therapy (ACT).

While ACT is frequently associated with modern oncology, its roots are deeply embedded in the management of infectious complications. A pivotal study in the 1990s established the clinical feasibility of this approach. Riddell et al. (1992) achieved a landmark success by using a Cytomegalovirus (CMV) -specific T cell therapy to treat life-threatening viral infections in bone marrow transplant recipients, the first successful ACT for a viral infection. Shortly thereafter, Rooney et al. (1995) demonstrated the efficacy of gene-modified cytotoxic T lymphocytes (CTLs) for Epstein-Barr virus (EBV), associated lymphoproliferative disorders [25–27]. These early milestones provided the proof of concept that ex vivo expanded lymphocytes could function as living drugs, seeking out and destroying infected cells in immunocompromised hosts. A subsequent milestone was the EU marketing authorization of tabelecleucel (Ebvallo) in December 2022, an allogeneic EBV-specific T cell immunotherapy for relapsed or refractory EBV-positive post-transplant lymphoproliferative disease (PTLD) following at least one prior therapy [28]; its clinical activity was subsequently reported in the phase III ALLELE study [29].

Despite these successes, traditional T cell therapies face limitations, including MHC restriction and the risk of Graft-versus-Host Disease (GVHD). This has prompted a search for more versatile effector cells. Among the candidates, including Natural Killer (NK) cells and γδT cells, cytokine-induced killer (CIK) cells represent a particularly promising novel approach. First discovered and characterized by Schmidt-Wolf et al. in 1991 [30] CIK cells are ex vivo expanded T lymphocytes (CD3^+^) that acquire the phenotypic and functional markers of NK cells (CD56^+^). They combine T-cell proliferative capacity with NK-like, non-MHC-restricted cytotoxicity, providing a rationale for their investigation in infectious diseases. Furthermore, seminal work by Schmidt-Wolf (1999) and Introna (2007) reported acceptable safety and low incidence of GVHD in allogeneic settings in early oncology trials which need to be established in infectious disease [31, 32].

The transformative success of cell-based immunotherapies in oncology, exemplified by CAR-T cells, has validated the power of immune engineering. However, infectious diseases remain recalcitrant due to challenges that mirror the tumor microenvironment: biofilm formation, latent or intracellular sequestration, immune evasion, multidrug-resistant, immunocompromised host settings, post-allogeneic hematopoietic stem cell transplantation (allo-HSCT) infections, climate and environmental challenges, and the continual emergence of novel pathogens. Extending the lessons learned from oncology to infectious diseases is a logical scientific progression. Recent milestones using immune cells along with ongoing trials against COVID-19, HIV, and tuberculosis signal that this translation is already underway.

**Fig. 2:**
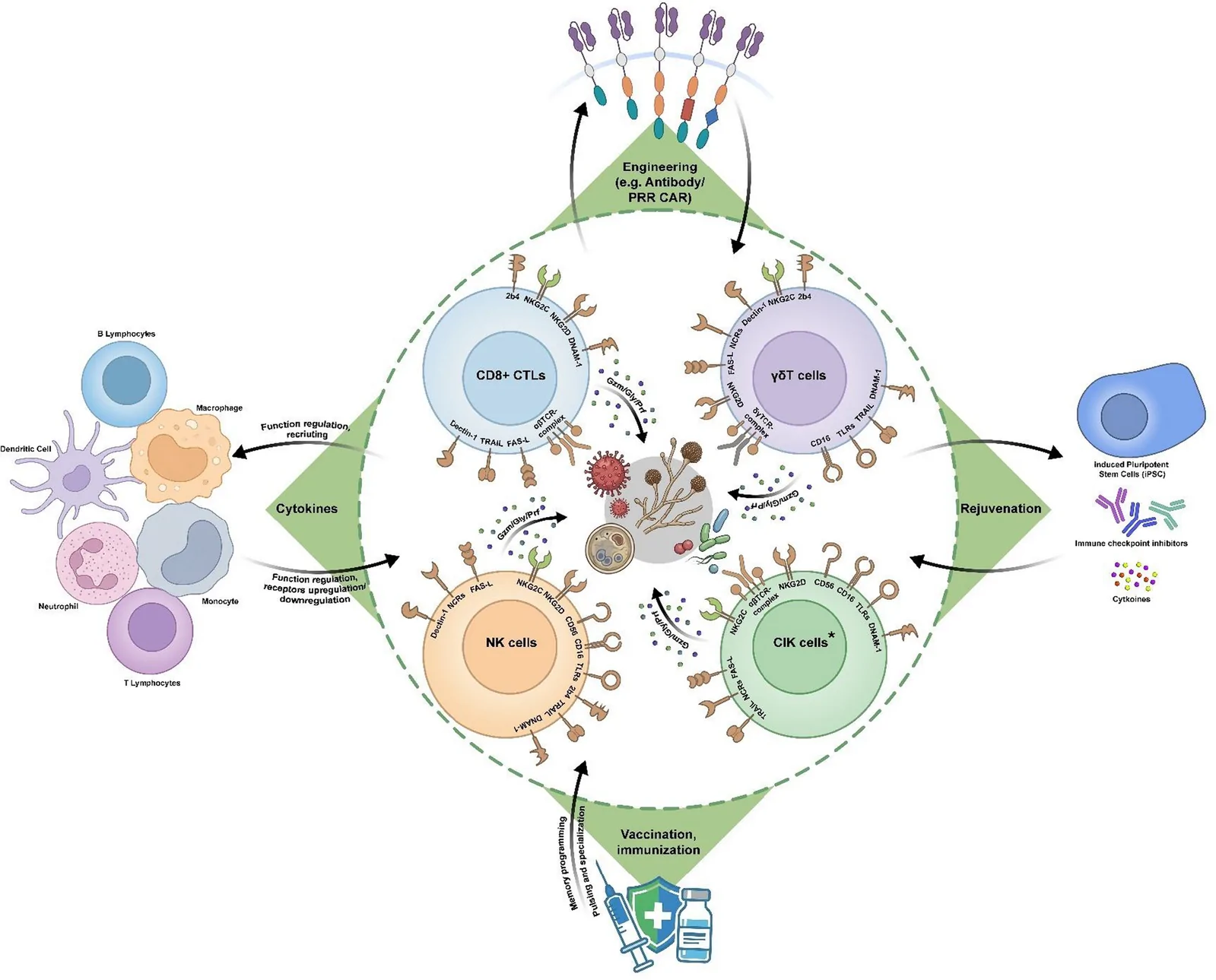
Convergent cytotoxic lymphocyte networks and translational modulation strategies in infectious disease immunotherapy. Schematic overview illustrating the shared and distinct cytotoxic mechanisms of CD8^+^ cytotoxic T lymphocytes (CTLs), γδT cells, natural killer (NK) cells, and cytokine-induced killer (CIK) cells in the context of infectious diseases; the experimental evidence supporting their proposed involvement in CIK activity is distinguished in Fig. 3; Table 2. All four effector populations employ overlapping cytolytic pathways, perforin-granzyme release, granulysin mediated antimicrobial activity, death receptor signaling (FasL and TRAIL), and antibody-dependent cellular cytotoxicity (ADCC), with similarities in antigen recognition strategies and receptor usage. CTLs primarily rely on T cell receptor (TCR)-mediated recognition of peptide-MHC complexes, whereas γδT cells and NK cells detect stress-induced ligands through MHC-independent mechanisms involving NKG2D, DNAM-1, natural cytotoxicity receptors (NCRs), and additional pattern-recognition receptors. CIK cells integrate adaptive and innate features, expressing TCRs alongside NK-associated activating receptors, most prominently NKG2D, enabling broad cytotoxic activity with minimal alloreactivity. Cytokines produced by cytotoxic lymphocytes shape local immune responses by recruiting and activating antigen-presenting cells and other innate and adaptive immune subsets. External modulatory strategies, including vaccination-driven immune priming, cytokine conditioning to enhance response quality, receptor upregulation for improved detection, and immune checkpoint downregulation, immune checkpoint inhibition, chimeric antigen receptor (CAR) engineering, and rejuvenation approaches such as induced pluripotent stem cell (iPSC)-derived immune cells can enhance cytotoxic function, persistence, and specificity. Together, the figure highlights CIK cells as a candidate cellular approach for further investigation that leverages shared cytotoxic programs across lymphocyte lineages and provides a framework for future cell-based interventions against infectious diseases. * The pathogen categories illustrated do not imply established CIK cell efficacy throughout each category; fungal applications remain proposed. The recognition pathways illustrated include mechanisms characterized in CIK oncology studies, pathways investigated in other cytotoxic lymphocytes during infection, and proposed extensions to CIK cells. Their inclusion illustrate the biological rationale for further investigation, with functional validation in infection-specific CIK experiments remaining necessary for proposed pathways

In this review, we examine the evolving landscape of cell-based immunotherapies for infectious diseases, placing a deliberate spotlight on CIK cells. We will explore their unique biology, summarize preclinical and clinical evidence of their antimicrobial efficacy, and propose future directions for evaluating CIK cell therapy in selected infectious settings. The convergent cytotoxic mechanisms, shared effector pathways, and potential translational strategies employed by CTLs, γδT cells, NK cells, and CIK cells are illustrated in Fig. 2.

## Cytotoxic T Lymphocytes (CTLs)

### Ontogeny, definition, and recognition mechanisms

Cytotoxic T lymphocytes (CTLs), traditionally identified as CD8^+^ T cells, represent a cornerstone of the adaptive immune system’s cellular defense network. These cells mature within the thymus through a rigorous selection process and subsequently circulate throughout the peripheral blood and lymphoid tissues, maintaining a state of vigilance against cellular aberrations. The foundational understanding of CTLs biology emerged in the 1960s and 1970s, primarily through investigations into the mechanisms of transplant rejection [33]. Their primary physiological mandate is to recognize and eliminate host cells that have been compromised by intracellular pathogens, including viruses, bacteria, fungi, and parasites, or have undergone malignant transformation [34]. This immunosurveillance function is indispensable for preventing disease progression and maintaining host homeostasis.

The defining characteristic of conventional CTLs is the expression of the CD8 co-receptor, which stabilizes the interaction between the T cell receptor (TCR) and class I major histocompatibility complex (MHC) molecules. MHC class I is expressed on nearly all nucleated cells, allowing CTLs to screen the internal proteome of tissues. The seminal discovery that pathogen recognition requires simultaneous engagement of the TCR and self-MHC molecules, a phenomenon known as MHC restriction, was first elucidated by Zinkernagel and Doherty in the context of viral infections [24]. Upon successful antigen engagement, coupled with appropriate co-stimulatory signals, naïve CD8^+^ T cells undergo a dramatic functional transformation. This initiates a cascade of signaling events leading to robust proliferation (clonal expansion) and differentiation into potent effector cells capable of immediate cytotoxicity, as well as memory subsets that provide long-term immunity [35].

### Phenotypic heterogeneity and specialized subsets

While the CD8^+^ lineage is often viewed as a monolithic population, it exhibits remarkable phenotypic heterogeneity. CTLs express a diverse repertoire of accessory receptors that fine-tune their activation thresholds, including the activating receptor NKG2C, the senescence marker CD57, and various killer-cell immunoglobulin-like receptors (KIRs) typically associated with NK cells [36]. For instance, specific environmental triggers such as CMV infection drive the expansion of a unique population of NKG2C^+^ CTLs [37].

Beyond the classical circulating pool, distinct subsets have evolved to address specific immunological niches. One such subset is the population of tissue-resident memory T cells (TRM). Distinguished by the expression of residency markers such as CD69, CD103, CD49a, and CD44, these cells do not recirculate but instead permanently inhabit peripheral non- lymphoid tissues [38]. Defined initially in the context of *Herpes simplex virus* (HSV) [39], they are particularly abundant at epithelial barriers, common portals of pathogen entry such as the skin, intestinal mucus, and reproductive tract. Their strategic positioning allows them to mount rapid, site-specific immune responses upon pathogen re- exposure, immediately releasing interferon-gamma (IFN-γ), granzyme B, and perforin without the delay required for recruiting circulating cells [38, 40, 41].

Furthermore, the classical dichotomy between CD4^+^ “helper” and CD8^+^ “killer” T cells is increasingly recognized as an oversimplification. Under specific inflammatory conditions, a subset of CD4^+^ T cells can acquire direct cytotoxic capabilities. These cytotoxic CD4^+^ T cells recognize antigens presented by MHC class Il molecules but possess a cytolytic machinery, including granzyme K. They establish residence in peripheral tissues and contribute to the clearance of infected cells, particularly in scenarios where CD8^+^ responses are compromised or exhausted. Their clinical relevance has been documented in infections caused by *Chlamydia trachomatis* and *Brucella abortus*, as well as in severe COVID-19. However, their efficacy appears context-dependent; for example, they show limited effectiveness against *Mycobacterium*-infected antigen presenting cells (APCs), suggesting pathogen-specific functional limitations [35, 42–45].

Another specialized subset is the Antimicrobial CTLs (amCTLs). These CD8^+^ T cells are characterized by the co-expression of cytolytic molecules including granzyme B, granulysin, and perforin with the activating receptor NKG2C [36]. Unlike conventional effectors, amCTLs possess the unique capacity to kill intracellular bacteria like *Listeria monocytogenes* and *Mycobacterium leprae* through mechanisms that can be either TCR-dependent or independent, providing a flexible layer of defense against pathogens that might otherwise evade detection [46].

Finally, HLA-E-restricted CD8^+^ CTLs represent a unique bridge between innate and adaptive immunity. These cells recognize peptides presented by HLA-E, a non-classical MHC class Ib molecule. HLA-E molecules can present viral peptides, such as CMV UL40, HIV p24, and *EBV* BZLF1 or their expression can be upregulated by these peptides. Unlike NK cells, which use HLA-E recognition primarily as an inhibitory signal, these CTLs recognize specific peptide-HLA-E complexes via their TCR, distinguishing them from NK cells [47]. This is evolutionarily significant because many viruses, such as HIV and CMV, downregulate classical MHC class I molecules to evade conventional CTLs. In CMV infection, the viral peptide UL40 mimics host leader sequences to stabilize HLA-E expression, thereby inhibiting NK cell attack. However, HLA-E-restricted CTLs can specifically detect these stabilized complexes and eliminate the infected cells [48]. Evidence suggests these cells also exhibit memory phenotypes, and similar recognition mechanisms have been observed *in Salmonella typhi* infection, where HLA- E presentation is necessary for lysis by vaccine-induced CTLs [49, 50].

### Mechanisms of cytotoxicity and effector function [35, 44–46, 49, 51–63]

To eliminate this broad array of threats, CTLs employ a sophisticated and diverse arsenal of cytotoxic mechanisms. Many of these pathways are evolutionarily conserved and shared with other cytotoxic lymphocytes, including NK cells, γδT cells, and cytokine-induced killer (CIK) cells.

The hallmark mechanism of CTLs cytotoxicity is the polarized release of lytic granules. These specialized secretory lysosomes contain a potent cocktail of effector proteins: perforin, granulysin, and a family of serine proteases known as granzymes (A, B, C, K, M). Perforin is a calcium-dependent, pore-forming protein that inserts itself into the target cell membrane, creating transmembrane channels. This polymerization event facilitates the entry of granzymes into the target cytosol. Once inside, granzymes initiate rapid apoptosis, primarily through the cleavage of caspase-3 and other substrates. Crucially, granulysin, a saponin-like lipid-binding protein, adds a unique dimension to this arsenal. Unlike perforin, granulysin can disrupt the membranes of bacteria and parasites or cholesterol deficient membrane directly, allowing CTLs to kill intracellular pathogens without necessarily destroying the host cell initially. This synergistic action is particularly vital for controlling robust intracellular dwellers like *Mycobacterium tuberculosis* (Mtb) and *Listeria*.

Parallel to granule exocytosis, CTLs can induce apoptosis via the Fas- Fas Ligand (Fas-L) pathway. The upregulation of Fas-L (CD178) on the activated CTLs surface allows binding to the death receptor Fas (CD95) on target cells. This engagement triggers the extrinsic apoptotic pathway via the activation of caspase-8, resulting in cell death independent of lytic granule contents.

Additional death receptor pathways contribute to CTL effector function. TNF-related apoptosis-inducing ligand (TRAIL) expressed by activated CD8^+^ T cells can promote target cell death; in a murine influenza model, TRAIL-DR5 interactions contributed to CD8^+^ T cell cytotoxicity and viral control. T cell-derived TNF can also induce target cell death through TNFR1-associated signaling, including RIPK1-dependent pathways demonstrated in experimental transplantation and tumor models. However, TNFR1 signaling can alternatively promote inflammation and cell survival, making its cytotoxic outcome dependent on the target cell signaling setting.

Recent research has unveiled an additional highly sophisticated killing mechanism: the secretion of supramolecular attack particles (SMAPs). These are autonomous, cytotoxic protein complexes approximately 120 nm in diameter. SMAPs consist of a glycoprotein shell encapsulating a core of granzyme B, granulysin, and perforin, stabilized by thrombospondin-1 (TSP-1) and serglycin. Once secreted, SMAPs can adhere to target cells and function as a “slow-release” cytotoxic system, remaining active even after the CTLs has physically detached from the target. This allows a single CTLs to deliver a lethal hit and immediately move on to the next target, enhancing the efficiency of killing.

Effectors are not limited to lytic mechanisms. In the context of HIV infection, CD8^+^ T cells have been shown to secrete a unique soluble factor known as the CD8^+^ antiviral factor (CAF). Remarkably, CAF inhibits viral replication by suppressing HIV gene transcription without inducing the lysis of the infected cell preserving the host tissue while halting viral spread.

Additionally, a distinct functional subset known as Tc17 cells secretes interleukin-17 (IL-17). While less cytolytic than Tc1 cells, Tc17 cells are critical for antifungal defense, promoting the recruitment of neutrophils to sites of infection and enhancing the innate immune response against pathogens like Blastomyces and Histoplasma.

### Cytokine orchestration and receptor modulation

Beyond direct killing, CTLs act as orchestrators of the immune microenvironment through the production of cytokines and chemokines. They are prolific producers of IFN-γ and tumor necrosis factor (TNF). These cytokines upregulate MHC expression on surrounding cells, enhancing antigen presentation, and recruit macrophages and other effector cells to the site of infection.

The differentiation and maintenance of CTLs are, in turn, governed by a complex cytokine milieu. Type I interferons (IFN-1) and interleukins such as IL-2, IL-6, IL-12, IL-18, and IL-21, secreted predominantly by CD4^+^ T cells and APCs, are essential for priming and functional tuning. IL-7 and IL-15 are particularly critical for the long-term survival and homeostatic proliferation of memory CD8^+^T cells. Furthermore, IL-4 derived from Th2 cells can modulate CTL function, shifting their primary cytotoxic mechanism from granule exocytosis toward the Fas-L pathway [35, 44, 45, 49, 53, 54, 56–61, 64–69].

The functional profile of CTLs is further regulated by the balance between activating and inhibitory receptors, which can, in turn, be fine-tuned by cytokines. In chronic infections like tuberculosis (TB), this balance is critical. NKG2C^+^ CTLs typically exhibit a potent cytolytic profile characterized by high levels of granzyme B, granulysin, and perforin. In contrast, CTLs expressing the inhibitory receptor NKG2A appear less cytotoxic, producing higher levels of IFN-γ, IL-10, and granzyme A and B, but lower IL-2 levels, suggesting a potentially regulatory or context dependent role when co-cultured with Mtb-infected macrophages. In patients infected with Mtb, a dysregulation of this balance has been observed. Mtb infection correlates with a skewed ratio favoring NKG2A^+^ cells over NKG2C^+^ cells, concurrent with a reduction in “triple-positive” CTLs (expressing perforin, granzyme B, and granulysin) or lower expression of granulysin and perforin. This receptor imbalance contributes to an impaired host defense [70]. In the context of CMV, NKG2C^+^CMV co-express granzyme B, granulysin, and perforin, indicating a highly cytotoxic phenotype capable of directly killing of CMV infected cells [37].

### Therapeutic potential: Evidence from preclinical and clinical studies

The pivotal role of CD8^+^ CTLs in controlling infection has been substantiated through extensive preclinical and clinical investigation across a spectrum of parasitic, bacterial, fungal, and viral diseases.

In the context of parasitic infections, CD8^+^ CTLs utilize specialized mechanisms to breach complex pathogen defenses. Research by Tiwari et al. demonstrated that in *Toxoplasma gondii* infection, CD8^+^ CTLs collaborate with macrophages to reduce the burden of brain cysts in the mouse. Crucially, this was not achieved by inhibiting tachyzoite growth, but through a perforin- dependent mechanism that physically penetrated and removed the cyst walls, allowing immune access to the dormant parasites. Granzyme B was highly expressed within these CTLs, supporting their lytic role [55]. Similarly, brain-resident CD8^+^ T cells have been shown to independently control latent *Toxoplasma* with functional heterogeneity through local antigen recognition [71]. In malaria models, CD8^+^ CTLs are essential for controlling both the liver and blood stages of the parasite [72]. In patients infected with *Plasmodium vivax*, CTLs were observed to eliminate HLA- expressing infected reticulocytes via a granulysin-dependent mechanism, notably independent of perforin [56]. Furthermore, it was mentioned that the quality of the T cell response is a determinant of outcome in *Trypanosoma cruzi* infection (Chagas disease); enhancing the frequency of early differentiated T cells and reducing inhibitory receptor expression correlates with superior parasite control [68].

In fungal infections, which are often lethal in immunocompromised hosts, CTLs play a surprisingly central role. Wang et al. identified that the downregulation of mTOR signaling is a key metabolic switch that enhances CD8^+^ CTL survival during lethal fungal sepsis, highlighting an immunometabolic axis for therapy [73]. In *Cryptococcus* infection, CD8^+^ CTLs contribute to clearance primarily through IFN-γ and granulysin, rather than perforin, and their depletion significantly worsens survival outcomes. The cytokine IL-15 has been shown to further augment this antifungal activity [59].

### Vaccination and induction of CTLs responses

Given their potency, the induction of robust CD8^+^ CTLs responses is a primary goal of next-generation vaccinology. Vaccine-induced CTLs have been consistently linked to reduced pathogen loads and improved survival [40, 74].

Advances in bioinformatics have enabled the creation of in silico-designed vaccines. For example, multi-epitope vaccines against cutaneous leishmaniasis have been engineered to specifically induce IFN-γ-producing CTLs [67, 75]. In the case of *Trypanosoma cruzi*, the N- Tc52/TSkb20 chimeric protein vaccine was shown to elicit robust CD8^+^ responses targeting fused epitopes, effectively reducing parasite burdens in critical organs [69]. DNA vaccination strategies have also yielded promising results; a DNA vaccine encoding the *Legionella pneumophila* antigen PAL92-100 induced strong antigen-specific CTLs that conferred 50% survival in lethal challenge models, a protection that was lost upon CD8^+^ depletion [66]. Similarly, Rotem et al. demonstrated that a DNA vaccine based on *Francisella tularensis holarctica* induced protective cellular immunity [76]. A bivalent vaccine using ghost cells of *Salmonella typhi* and *Salmonella paratyphi* stimulated both humoral and cellular responses, including CD8^+^ CTLs [77]. In the *Plasmodium berghei* malaria model, the specific peptide SYIPSAEKI from the circumsporozoite protein (CSP) was identified as a critical epitope for eliciting the CD8^+^ CTLs necessary for killing liver-stage parasites [72, 78]. Live-attenuated *herpes simplex virus-1 (HSV-1)* vaccines have demonstrated the ability to trigger robust CD8^+^ T cell responses, resulting in enhanced antiviral protection [54]. In cases of lethal fungal pneumonia, CD8^+^ CTLs play a pivotal role, even in the absence of CD4^+^ T cells. Vaccination-induced lL-17-secreting CD8^+^ CTLs are particularly crucial in combating *Blastomyces dermatitidis* and *Histoplasma capsulatum* infections, primarily by promoting neutrophil recruitment to the infection site [59].

Vector-based delivery systems are particularly effective at priming class I restricted responses. *Listeria monocytogenes*, itself a potent inducer of cytosolic antigen presentation, has been utilized as a vector to deliver *Coxiella burnetii* peptides. This strategy elicited strong CD8^+^ CTLs responses and elevated IFN-γ levels, reducing load of bacteria and conferring protective immunity upon adoptive transfer to naive hosts [79]. In viral models, adenoviral vectors delivering *Influenza* proteins have been shown to generate high-quality and frequency CTLs that determine heterosubtypic immunity, protecting against mismatched viral strains [80].

The importance of CTLs in vaccine mediated protection extends to COVID-19. Individuals with hybrid immunity (prior infection followed by mRNA vaccination) exhibit CD8^+^ T cells enriched for antiviral gene expression and cytolytic potential, offering superior protection compared to vaccination or infection alone. Notably, HLA variation affects the efficiency of these responses, suggesting that the stability of the peptide-MHC-TCR interaction is a critical determinant of viral evasion or control [81].

Together, these findings demonstrate that vaccine induced CTLs are not only indispensable across diverse infectious contexts, but also can shape the quality and quantity of responses by indirect engineering of CTLs.

### Adoptive cell therapy (ACT) and genetic engineering

Beyond active vaccination, the direct ACT of ex vivo expanded CTLs offers an immediate prevention and therapeutic intervention [82]. This approach has matured significantly in the treatment of viral complications following allo-HSCT. A comprehensive meta-analysis of 40 studies involving 953 allo-HSCT recipients confirmed the efficacy of donor derived or third party CMV-specific CTLs, reporting an overall response rate of 90.16% and a complete response rate of 82.59% with an excellent safety profile [83, 84]. In the context of COVID-19, Grosso et al. demonstrated that the transfer of third-party, virus-specific CD8^+^ CTLs persisted for at least 6 months and led to a > 99% reduction in viral load within 14 days in HLA-A*02:01-matched patients, without causing alloimmunization or GVHD [85].

Innovative engineering strategies are further expanding the utility of CTLs. For HIV cure research, adoptive transfer of vaccine-primed, central memory CD8^+^ CTLs into SHIV-infected rhesus macaques successfully suppressed viral rebound after the interruption of antiretroviral therapy (ART) [86]. A futuristic approach involves the “rejuvenation” of exhausted HIV-specific CTLs by reprogramming them into induced pluripotent stem cells (iPSCs) and re-differentiating them back into CTLs. These rejuvenated cells exhibit extended telomeres and enhanced HLA- restricted HIV suppression in vitro compared to their original CTLs counterparts [87].

Finally, chimeric antigen receptor (CAR) T cell technology, famous for its success in leukemia, lymphoma, and multiple myeloma, is also being explored for infectious diseases. Human clinical investigation has been undertaken in HIV-1 infection. Liu et al. evaluated broadly neutralizing antibody-derived CAR-T cells in 14 participants, reporting acceptable tolerability and reductions in viral reservoir measures; however, viral rebound occurred in all six participants who interrupted ART. More recently, a multicenter phase I study evaluated M10 CAR-T cells in combination with chidamide in 18 participants with HIV-1, providing further clinical experience with this approach. These findings support clinical feasibility, while durable ART-free viral control remains unestablished in these studies [88, 89]. In preclinical studies, *Aspergillus fumigatus*-specific CAR-T cells have been engineered to directly target fungal elements via perforin and granzyme B while simultaneously augmenting innate response by activating macrophages through cytokine release [58]. Similarly, Dectin-1- based CAR-T cells, which target the conserved β-glucan carbohydrate found in fungal cell walls, successfully reduced hyphal growth in mouse models via perforin/ granzyme release and IFN-γ production [60]. In the bacterial realm, genetically engineered F5 TCR-transgenic CD8^+^ CTLs designed to target *Staphylococcus aureus*-infected dendritic cells have demonstrated the ability of antigen-specific IFN-γ production and cytotoxicity against reservoirs of infection, providing preclinical evidence rather than a demonstration of therapeutic efficacy in patients [90].

Complementing these cell-based approaches, reducing inhibitory receptors or immune checkpoint inhibitors targeting CTLA-4 and PD-1, originally developed for cancer, have shown efficacy in murine models of pulmonary paracoccidioidomycosis, prolonging survival by reversing T cell exhaustion [68, 91].

Collectively, these diverse biological insights and therapeutic innovations, ranging from the molecular precision of peptide vaccines to the cellular engineering of CAR-T and iPSC derived cells, underscore the pivotal role of cytotoxic T lymphocytes. They represent a powerful, flexible, yet complex platform that forms the backbone of modern immunotherapeutic strategies against infectious diseases.

## Gamma delta (γδ) T cells

### Ontology, genomic architecture, and distinct developmental lineage

Gamma-delta (γδ) T cells constitute a phylogenetically conserved, distinct lineage of T lymphocytes that function as a critical bridge between the innate and adaptive immune systems. Unlike conventional αβ T cells, which recognize processed peptide antigens presented by MHC molecules, γδT cells have evolved a unique “dual-receptor” biology that allows them to function largely independently of MHC restriction. First identified in the 1980s by Susumu Tonegawa and colleagues during the search for the TCR α chain, the discovery of the TCR δ chain gene rearrangement challenged the prevailing binary paradigms of immunology [92, 93]. γδT cells originate from common lymphoid progenitors in the bone marrow but diverge from the αβ lineage early in thymic development. While they share the thymus as a primary site of maturation, their developmental checkpoints are distinct; the majority of γδT cells exit the thymus as double-negative (CD4^−^CD8^−^) effectors. Approximately 30% of the peripheral population expresses CD8, typically as CD8αα homodimers rather than the CD8αβ heterodimers found on conventional CTLs, and a negligible fraction expresses CD4 [94, 95]. This distinct phenotype reflects their specialized role not just as helpers or killers, but as autonomous immunologic sentinels capable of rapid, independent action.

### Receptor biology: The integration of innate and adaptive signals

The functional capacity of γδT cells is defined by their ability to integrate signals from a somatically recombined TCR and a vast array of germline-encoded innate receptors. The γδTCR exhibits broad specificity, functioning similarly to a pattern recognition receptor (PRR) but with the clonal diversity of an adaptive receptor. It is capable of detecting a diverse array of fundamental biochemical signatures, including haptens, small pyrophosphate metabolites, neurotransmitters, microbial products, post-translationally modified proteins, and host-derived molecules. This versatility is key to their surveillance of microbiome-derived antigens, which support immunological homeostasis and rapid effector function upon activation [96–98]. Crucially, this TCR-mediated sensing is complemented, and often amplified, by a potent arsenal of innate receptors typically associated with NK cells. γδT cells respond to various MHC-independent ligands, such as β-glucans, *Influenza* hemagglutinin, HSV-1 glycoprotein I, heat-shock proteins, and F1 ATPase. Notably, they can directly recognize *Malassezia*-derived ligands without the need for antigen presentation by APCs. All these capabilities arise through expressing wide ranges of receptors including the Fc receptor CD16 (FcyRIll), the adhesion molecule CD56, activating receptors NKG2C and NKG2D, the co- stimulatory receptor 2B4, DNAM-1, the natural cytotoxicity receptors NKp30, NKp44, and NKP46, Dectin-1, and TLRs [99–106]. Among these, NKG2D serves as a master regulator of stress surveillance. It recognizes MHC class I chain-related proteins A and B (MICA/B) and UL16-binding proteins (ULBPs) in humans (and Rae-1, H60, MULT1 in mice) [107, 108]. Under homeostatic conditions, these ligands are barely detectable. However, their expression is dramatically upregulated in response to DNA damage, oxidative stress, viral replication (e.g., CMV, HCV), *Mycobacterium* infection, sepsis, or malignant transformation, acting as a distress beacon to the immune system [109, 110]. Although MICA/B structurally resemble MHC class I molecules, they do not present peptides and instead serve purely as stress signal. Consequently, this pathway is frequently targeted by pathogen evasion strategies. In chronic pathologies such as *Hepatitis B Virus* (HBV), COVID-19, and advanced sepsis, the proteolytic shedding of MICA results in high serum levels of soluble MICA (sMICA). These soluble ligands bind to NKG2D on circulating lymphocytes, inducing receptor internalization and degradation, thereby creating an immune blind spot [111–113]. Similarly, viruses such as HCV, HHV, and CMV have evolved mechanisms to mask ULBPs or retain them in the endoplasmic reticulum to evade detection [114–116]. Nevertheless, when this system functions correctly, the simultaneous engagement of the TCR and NKG2D creates a synergistic activation threshold. For instance, in *Brucella*-infected macrophages expressing ULBPs and in Mtb-infected cells expressing MICA, NKG2D engagement enhances TCR dependent γδT cell cytotoxicity and cytokine production, inhibiting bacterial proliferation more effectively than either receptor could achieve in isolation [100]. Furthermore, the engagement of NKp30 on γδT cells triggers the release of β- chemokines such as MIP-1α, MIP-1β, and RANTES. These chemokines bind to CCR5, competitively blocking the entry of CCR5-tropic HIV-1 strains into target cells, illustrating a sophisticated non-cytolytic mechanism of viral control [117].

### Functional plasticity: The type 1 and type 17 paradigms

Upon activation, γδT cells unleash a pleiotropic effector program that includes antibody-dependent cellular cytotoxicity (ADCC) via CD16, phagocytosis, the release of cytotoxic granules (containing granzymes A/B, granulysin, and perforin), and the engagement of death receptors such as Fas-L and TRAIL [100–102, 104, 105, 117–125]. They also secrete a range of cytokines such as IFN-γ, TNF, IL-4, IL-10, IL-17, IL-18, IL-22 and chemokines like CCL3, CCL4, CCL5 to orchestrate immune recruitment such as neutrophils [96, 99, 100, 104, 117, 119, 120, 124, 126, 127]. Moreover, IL-2, IL-15, and IL-18 enhance γδT cell expansion and increase expression of activation receptors such as NKp30 and NKp44 in Vδ1 cells [100, 104].

Beyond these, they are prolific producers of cytokines exhibiting a functional plasticity that is often pre-programmed during thymic development in a mechanism distinct from the peripheral polarization of Th1/Th2 cells [128]. This dichotomy is broadly categorized into Type 1 and Type 17 responses.

The Type 1 response is characterized by the robust secretion of IFN-γ and TNF, driving nonlytic cytotoxicity against intracellular pathogens and viruses [104]. In the context of *Staphylococcus aureus* infection, IFN-γ produced by γδT cells is critical for orchestrating macrophage activation and neutrophil recruitment. However, studies indicate that this secretion is strictly dependent on the presence of IL-12 and direct cell-to-cell contact with infected cells [127]. In *Influenza* infection, γδT cell-derived IFN-γ plays a protective role by directly inhibiting viral replication and promoting the influx of other immune effectors [117]. Conversely, in chronic HCV infection, although γδT cells exhibit elevated activation markers, their capacity for IFN-γ production is often functionally exhausted or impaired. Paradoxically, the administration of therapeutic IFN-α in these settings has been reported to exacerbate this impairment, leading to liver immunopathology without restoring effective viral clearance [102].

The Type 17 response is essential for mucosal barrier immunity and defense against extracellular pathogens, particularly fungi and extracellular bacteria. The production of IL-17 plays a crucial role not only in mucosal and skin immunity against parasites like *Trypanosoma* but also in the immune response to fungi such as *Candida albicans*. Upon exposure to *Candida albicans*, the direct activation of the C-type lectin receptor Dectin-1 on Vδ1 T cells induces a rapid release of IL-17 cytokine [99, 129, 130]. This axis is often amplified by IL-23 and IL-1β secreted by dendritic cells, creating a feed-forward loop that enhances antifungal immunity [99, 129]. In *Helicobacter pylori* infection, elevated secretion of IL-17 and IL-22 by γδT cells contributes to the maintenance of mucosal epithelial barrier integrity and the recruitment of neutrophils [131, 132]. During *Influenza* infection, chemokines secreted by respiratory epithelial cells (CCL3, CCL4, CCL5) recruit CCR5^+^ γδT cells to the infected lungs, where they produce IL-17 to promote neutrophil and NK cell recruitment, facilitating tissue repair and providing protection [117]. However, this potent inflammatory response functions as a double-edged sword; in tuberculosis, while both IFN-γ and IL-17 are required for containment, dysregulated IL-17 production can drive excessive neutrophil infiltration, granuloma necrosis, and tissue damage [93, 133].

### The Vδ1 subset: Sentinels of tissue integrity

γδT cells are taxonomically classified based on their TCR δ-chain usage, which dictates their anatomical localization and ligand specificity. Vδ1 T cells, predominantly paired with various γ-chains, constitute the major tissue-resident population. They localize to epithelial interfaces such as the gut, mucosa, skin, liver, spleen, and the female reproductive tract, functioning as the first line of immunological defense [102, 104]. Their tissue residency in the lung during Mtb infection and higher expansion in spleen in malaria-endemic regions further indicates their non-circulatory location and pathogen-specific recruitment [134, 135]. Unlike their Vδ2 circulating counterparts, Vδ1 cells possess a diverse TCR repertoire with extensive CDR3 junctional complexity, reflecting an adaptive-like capacity for specific antigen selection and clonal expansion [134, 136].

These cells recognize a unique spectrum of antigens, including lipid antigens presented by CD1 molecules through their γδTCR, independently of classical MHC, and stress ligands via NKG2D and TCR [93, 137, 138]. Their role is particularly pronounced in chronic viral infections and tissue surveillance. In CMV infection, Vδ1 cells undergo massive clonal expansion and differentiate into long-lived, memory-like effectors (CD45RA^+^CD27-) capable of controlling viral reactivation, demonstrating that they can mount antigen-driven adaptive responses distinct from classical T cell memory [100, 136]. In HIV infection, Vδ1 cells in the gastrointestinal mucosa, a key reservoir of viral persistence, express high levels of CD8, CD56, CD16, NKp30, and NKG2D. These cells retain potent cytotoxic potential even during ART suppression, and their antiviral activity can be further boosted by IL-15 pretreatment, suggesting a therapeutic avenue to target HIV reservoirs [139]. Interestingly, Vδ1 cells in HIV patients also exhibit upregulated NKG2C expression, mirroring the phenotype of adaptive NK cells [117]. In the context of fungal co-infection, *Candida albicans* exposure in HIV patients triggers a Vδ1 expansion more than mono-infected patients dependent on direct contact with dendritic cells and the secretion of IL-18, IL-6, and IL-23 [99].

However, some chronic antigen exposure can lead to functional exhaustion. In a study of placental malaria, Nana et al. documented significantly increased frequencies of Vδ1 cells in both maternal blood and intervillous blood mononuclear cells (IVBMC). These cells exhibited high expression of exhaustion markers such as PD-1 and TIM-3, which correlated with adverse pregnancy outcomes like low birth weight and maternal anemia. This suggests that while Vδ1 cells expand in response to the parasite, their protective function becomes compromised in the highly regulated environment of the placenta [140]. Despite expressing checkpoints like PD-1 and TIGIT, specific subsets of Vδ1 cells (CD8^+^CD16^+^CD56^+^) often retain granzyme-independent cytotoxic functions highlighting a resilience in their effector programming even in chronic disease states [101].

### The Vγ9Vδ2 subset: Sensors of metabolic stress

The Vγ9Vδ2 T cell subset (often referred to as Vδ2) dominates the peripheral blood of humans and non-human primates. These cells secrete low levels of IL-17 but exhibit higher levels of IFN-γ production, with their frequency notably declining with age, especially in women over 40 [101, 102, 127]. Compared to Vδ1 cells, Vδ2 T cells display elevated CD3 zeta chain expression, enhancing their potent effector functions, although they demonstrate slower activation kinetics, potentially due to reduced sensitivity to ionomycin [101]. Nevertheless, Vδ2 T cells can rapidly activate in response to *Staphylococcus aureus*-infected dendritic cells [127]. Functionally, they produce significantly higher levels of cytotoxic molecules, such as granzyme B and perforin, compared to Vδ2^−^ cells [101, 141].

These cells function as innate-like sensors of metabolic dysregulation. Although they can interact with HLA-E and ULBPs [137], their hallmark feature is the recognition of small pyrophosphate metabolites, known as phosphoantigens (pAgs), through there γδTCR. These include isopentenyl pyrophosphate (IPP), a metabolic intermediate of the mammalian mevalonate pathway, and (E)-4-hydroxy-3-methyl-but- 2-enyl pyrophosphate (HMBPP), a highly potent metabolite produced by tumor cells, bacteria (e.g., *Mycobacterium*, *Listeria*), and parasites (e.g., *Plasmodium*) [100, 104].

The mechanism of this recognition is sophisticated and involves the transmembrane protein Butyrophilin 3A1 (BTN3A1) and its partner BTN2A1. Intracellular accumulation of pAgs induces a conformational change in the intracellular B30.2 domain of BTN3A1. This conformational shift is transmitted to the cell surface, recruiting the Vγ9Vδ2 TCR to the complex [100, 104, 119, 126, 127]. This “inside-out” signaling mechanism enables Vδ2 cells to detect intracellular pathogens such as Mtb, *Listeria monocytogenes*, *Coxiella burneti*, and *Toxoplasma gondii* without requiring antigen processing or MHC presentation [123, 142, 143]. This detection translates into potent effector function; for instance, Vδ2 T cells exhibit robust cytotoxicity against intracellular *Brucella abortus* via the granule-mediated killing of infected macrophages. This response is characterized by the upregulation of granzyme B, CD107a, and CD25, alongside IFN-γ and TNF production, which effectively suppresses bacterial replication in both murine and bovine models [124]. Levels of BTN3A1 are notably elevated in various infections, including malaria, COVID-19, and EBV, serving as a beacon for immune recognition [119, 122, 144].

Microbial p-Ags, such as HMBPP, combined with cytokines like IL-2 and IL-21, are widely recognized as an effective method for the ex vivo activation of Vδ2 T cells, enabling them to upregulate activation markers such as CD16, CD25, and CD69 as well as cytolytic effectors. Additionally, endogenous p-Ags pathway is clinically exploitable using aminobisphosphonates like Zoledronate (ZOL) or Pamidronate (PAM). These drugs inhibit the enzyme farnesyl pyrophosphate synthase (FPPS) in the mevalonate pathway, leading to the upstream accumulation of IPP in monocytes and tumor cells, which subsequently triggers massive Vδ2 activation [145–147]. In pre-clinical models of tuberculosis, ZOL plus IL-2 therapy induced the expansion of Vδ2 cells producing IFN-γ and perforin and enhancing NK cells, CD4^+^, CD8^+^ T cells responses while decreasing exhausted cells which correlated with reduced bacterial burdens and improved lesion healing in macaques [148, 149]. Similarly, clinically in HIV infection, ZOL plus IL-2 therapy can reinvigorate Vδ2 responses, improve their ADCC cytotoxicity, and indirectly boost HIV- specific CD8^+^ T cells via dendritic cell maturation [145, 150, 151]. On the other hand, PAM/IL-2 activated Vδ2 T cells differentiate into a potent effector phenotype expressing CD69, CD25, HLA-DR, CD8, CD56, and CD16, alongside elevated levels of the degranulation marker CD107a. These activated effectors elicit robust humoral and cellular immunity through significant expansion and pro- inflammatory cytokine production. Crucially, they possess the unique ability to recognize and destroy latently infected CD4^+^ T cells, addressing a major hurdle in HIV eradication strategies [152]. Furthermore, PAM and IPP-activated Vγ9δ2 T cells demonstrate strong antiviral activity against *Influenza* virus infections both in vitro and in vivo, albeit less potent than ZOL in certain contexts. Depending on the viral strain, this control mediated through either direct cytotoxicity or high-level IFN γ secretion [104, 151]. However, like Vδ1, impairments in Vδ2 functionality have been noted in active tuberculosis and certain viral or parasite diseases, where cells may become anergic or depleted due to chronic overstimulation [126, 153].

In the context of COVID-19, Gay et al. reported a depletion of Vγ9Vδ2 cells in the peripheral blood of patients, likely due to recruitment to the infected lungs. These cells recognize BTN3A1, which is upregulated on infected lung epithelial cells. Upon activation, they infiltrate the tissue and execute potent cytotoxicity via degranulation and IFN-γ as well as TNF secretion. Crucially, these effects could be significantly enhanced in vitro by stimulation with agonistic anti-BTN3A antibodies, suggesting a potential therapeutic avenue to restore antiviral immunity [119].

Against parasites, Vγ9Vδ2 cells expand and mature into cytotoxic effectors expressing high levels of granzyme B and perforin during congenital *Toxoplasma gondii* infection, responding directly to parasite-derived HMBPP [123]. In *P. falciparum* malaria, Vγ9Vδ2 cells proliferate in the peripheral blood, express activation markers like CD69 and CD16, and release granzyme B and granulysin upon contact to kill parasites [122, 154–156]. However, repeated exposure in endemic regions, coupled with aging, can result in a decline in cell numbers and functional exhaustion, evidenced by diminished cytokine production [153].

Furthermore, Vδ2 T cells demonstrate direct fungicidal activity against azole-resistant *Aspergillus fumigatus* hyphae and other mucormycetes such as *Cunninghamella bertholletiae* and *Rhizopus microspores*. This killing is primarily mediated by degranulation following direct cell-to-cell contact, as evidenced by CD107a upregulation, rather than through soluble factors alone [118].

### Minor subsets: Vδ3 and Vδ5

While Vδ1 and Vδ2 cells dominate the literature, other subsets such as Vδ3 and Vδ5 play specialized roles. These populations are rare in peripheral blood but are often enriched in the liver. Notably, Vδ3 and Vδ5 T cells have been shown to recognize specific host molecules such as the Endothelial Protein C Receptor (EPCR) and Annexin A2, independent of MHC or CD1, expanding the surveillance capacity of γδ compartment beyond classical stress ligands [100, 157, 158].

### Less well-known effector mechanisms: Phagocytosis and APC function

Beyond conventional cytotoxicity mechanisms already discussed, γδT cells possess “myeloid-like” capabilities that distinguish them from conventional lymphocytes and further underscore their evolutionary role as a bridge between innate and adaptive systems.

First, they are capable of professional antigen presentation. Activated Vγ9Vδ2 cells upregulate MHC Class II (HLA-DR) and costimulatory molecules (CD80, CD86 CCR7), allowing them to process and present *Plasmodium* and *Listeria monocytogenes* antigens to conventional CD4^+^ and CD8^+^ T cells [104, 159, 160]. This function is particularly effective when induced by *Staphylococcus aureus* or *Mycobacterium bovis* infected dendritic cells, where Vδ2 cells facilitate dendritic cell maturation via IL-12 and TNF loops, ultimately leading to a more effective adaptive T cell response [104, 127].

Second, γδT cells exhibit phagocytic activity. They are capable of engaging in actin-dependent phagocytosis, as demonstrated by their ability to internalize *E. coli* through opsonization and interaction with CD16 [161]. In *P.* falciparum malaria, Vγ9Vδ2 cells form immunological synapses with infected RBCs and actively phagocytose the parasites in a CD16-dependent manner [122, 154–156]. This is not merely a clearance mechanism; as discussed earlier, the expression of MHC class Il on these cells enables the cross-presentation of the phagocytosed pathogen-derived antigens to adaptive T cells, effectively priming the broader immune system against the infection.

### Therapeutic potential and future directions

The versatility of γδT cells makes them attractive candidates for immunotherapy in immunocompromised settings, including after allogeneic HSCT. In pediatric TCR αβ/CD19-depleted haplo-HSCT, early higher reconstitution of Vδ2 T cells is associated with reduced CMV reactivation and is accompanied by enhanced CMV-specific αβ T cell responses in vitro, highlighting their direct and bystander antiviral functions. Furthermore, multiple HSCT cohorts have shown that robust γδT cell reconstitution correlates with fewer infections and improved event-free and overall survival [162, 163]. Their ability to function as a universal donor cell type due to their lack of MHC restriction reduces the risk of GVHD, making them safer for allogeneic transfer than conventional T cells. In another open-label pilot study, eight patients with multidrug-resistant tuberculosis (MDR-TB) received 12 biweekly infusions of allogeneic Vγ9Vδ2 T cells (1 × 10^8^ cells) as adjunctive therapy. The treatment was well-tolerated with no significant toxicity and led to marked radiological improvement and weight gain. Immunologically, infusions promoted NK cell maturation and expanded effector/memory T cell subsets. Crucially, 7 of 8 patients achieved sustained sputum culture conversion, yielding an estimated cure rate of 87.5% when combining Vγ9Vδ2 cell therapy with anti-MDR-TB drugs, compared to 54% with just standard pharmacotherapy, suggesting this cell therapy effectively enhances bacterial clearance and lesion repair in MDR-TB [164].

Moreover, the memory-like features of Vδ2 T cells hold promise for vaccine development. BCG vaccination or exposure to *Staphylococcus aureus*-infected dendritic cells can trigger memory-like expansion characterized by CD45RO expression, enhancing functional readiness upon re-challenge [93, 101, 127, 145]. Additionally, γδT cells demonstrate broad antibacterial activity against a range of pathogens including *Streptococcus pneumoniae* [165], and *Klebsiella pneumoniae* [166], as well as playing roles in HIV/TB co-infection dynamics [167].

In summary, the γδT cell compartment represents a potent, dual-functional arm of the immune system. From the tissue-resident surveillance of Vδ1 cells to the phosphoantigen-sensing power of circulating Vδ2 cells, they occupy a unique functional niche. Their ability to kill via MHC-unrestricted mechanisms, secrete directing cytokines, and even perform phagocytosis positions them as a complementary force to conventional T cells and a logical target for next-generation cell-based therapies.

## Natural killer (NK) cells

### Ontogeny, education, and biology of NK Cells

Natural killer (NK) cells, first identified in the late 1960s and early 1970s, represent a critical lineage of innate lymphoid cells. They were initially designated as “null killer” cells due to their unique capacity to lyse target cells without the requirement for prior antigen sensitization or major histocompatibility complex (MHC) restriction, distinguishing them from the adaptive T and B lymphocyte compartments [168]. As a frontline defense component of the innate immune system, NK cells are indispensable for the rapid identification and elimination of intracellular pathogens, particularly viruses as well as the surveillance of malignant transformation and the removal of senescent or stressed cells. While morphologically resembling large granular lymphocytes, NK cells are functionally distinct, operating through a germline-encoded recognition system that detects “missing self” (loss of MHC class 1) or “induced self” (upregulation of stress ligands).

The maturation of human NK cells is a complex, stepwise process that occurs primarily in the bone marrow and secondary lymphoid tissues, where they acquire functional competence through a process termed “education” or “licensing.” During this developmental window, NK cells interact with self-human leukocyte antigen (HLA) ligands via their inhibitory receptors, particularly through KIR receptors and NKG2A. This interaction does not silence the cell but rather tunes its responsiveness; NK cells that engage self-MHC with sufficient affinity are licensed to become functionally potent effectors, ensuring they remain tolerant to healthy autologous tissue while possessing a lowered activation threshold for targets lacking these self-molecules. This education process is phenotypically mirrored by the transition from the immature, immunoregulatory CD56^bright^ subset to the mature, highly cytotoxic CD56^dim^ population. The latter subset progressively acquires KIR receptors, the C-type lectin receptor CD94/NKG2A, the maturation marker CD57, and high levels of cytotoxic effector molecules such as perforin and granzyme B. Furthermore, the upregulation of CD16 (FcyRllla) on mature NK cells confers the ability to mediate ADCC, thereby bridging the innate and adaptive humoral responses. This education ensures NK cells achieve self-tolerance while retaining the capacity to eliminate non-self and stressed cells amid diverse infections [169, 170].

Although conventionally defined as CD3^−^CD56^+^ lymphocytes, the NK compartment is highly heterogeneous. The CD56^bright^CD16^dim^ subset, which constitutes a minor fraction of NK cells in peripheral blood but predominates in secondary lymphoid organs, is largely responsible for cytokine production, specifically IFN-γ and TNF, and immune regulation. In contrast, the circulating CD56^dim^CD16^bright^ subset predominates and further maturates in direct cytotoxicity by acquisition of KIRs and terminal differentiation markers such as CD57. Notably, NK cell receptor expression is not static; it dynamically adjust during chronic infections affected by environment, involving the coordinated upregulation or downregulation of activating and inhibitory receptors that directly alters NK cell functionality [171–175]. Chronic pathogenic capability can significantly reshape this landscape; viral infections often drive the accumulation of terminally differentiated NK cells (e.g. CD56^dim^CD16^dim^, CD57^+^CD16^+^ NK cells) or expansion of unique subsets, such as CD56^−^ NK cells in HIV and malaria or memory-like populations in CMV infection. Detailed high-dimensional flow cytometry has further resolved this heterogeneity, identifying other subsets such as tissue resident NK cells (CD69^+^CD103^+^CD49a^+^CD56^bright^CD16^dim^) that possess distinct homing and functional capacities tailored to specific organ environments [176–182].

### Receptor signaling, cytotoxicity, and immune crosstalk

The activation of NK cells is governed by the dynamic integration of opposing signals derived from a sophisticated repertoire of germline-encoded receptors. Cytotoxicity is triggered when activating signals outweigh inhibitory inputs. Key activating receptors include the natural cytotoxicity receptors (NKp30, NKp44, NKp46), NKG2D, Dectin-1, DNAM-1, TLRs, and activating KIRs. These receptors recognize a diverse array of ligands, such as viral hemagglutinins, fungal cell wall components, and stress-induced molecules (e.g., MICA/B, ULBP) that are upregulated on infected or transformed cells. Conversely, inhibitory receptors, primarily inhibitory KIRs and the CD94/NKG2A heterodimer, recognize classical and non-classical MHC class I molecules. Under homeostatic conditions, the engagement of inhibitory receptors by self-MHC recruits tyrosine phosphatases (SHP-1/SHP-2) to the immune synapse, dampening activation signaling, preventing autoimmunity and provide a fail-safe mechanisms to avoid unwanted damage to healthy cells. However, in the context of viral infection or malignancy, the downregulation of MHC class l, a common immune evasion strategy, removes this “brake,” allowing the “accelerator” signals from stress ligands to dominate, resulting in target lysis. A good example in this context are NK responses against fungal infection which have gained increasing recognition, extending beyond simple surveillance to direct fungicidal activity. Interactions between NK cells and fungi are mediated largely by the NCR family. NKp30 has been identified as a critical activating receptor that directly recognizes fungal cell wall components, including β-1,3-glucan, on pathogens such as *Cryptococcus* and *Candida albicans*. Clinical observations support this mechanistic link, as HIV-infected individuals with reduced surface expression of NKp30 exhibit significantly impaired NK-mediated killing of *Cryptococcus*, which can be partially restored by IL-12 stimulation. Furthermore, expanded subsets of NK cells (CD56^+^CD3^−^NKG2D^+^NKp30^+^) have demonstrated potent activity against the swollen conidia of *Aspergillus fumigatus* mediated primarily through perforin dependent degranulation and involving Dectin-1 mediated recognition of β-glucans [183–186]. In the context of *Aspergillus fumigatus*, the interaction is highly specific; the hyphal cell-wall polysaccharide galactosaminogalactan (GAG) serves as a ligand for CD56 (NCAM-1) itself. The engagement of CD56 by GAG triggers robust NK activation, resulting in the secretion of granzyme B, perforin, IFN-γ and a specific profile of chemokines including CCL3, CCL4, and CCL5. These chemokines recruit additional innate effectors to the site of infection, amplifying the antifungal response [185, 187].

NK cells execute their effector functions through distinct mechanisms; the directed exocytosis of lytic granules containing granzymes, granulysin, and perforin which induce lysis and caspase-dependent apoptosis and the expression of death receptor ligands such as Fas-L and TRAIL. They can also mediate phagocytosis, and ADCC through IgG recognition via CD16 [171–173, 183, 188–200]. A novel mechanism of bacterial control was elucidated by Radomski et al. during *Chlamydia psittaci* infection. This study identified that NK cells are capable of internalizing bacteria and routing them into lytic granules. Within these organelles, the bacteria are processed by granzyme B, rendering them non-infectious yet highly immunogenic. Upon subsequent degranulation, this processed bacterial material is released and can induce a robust, protective Th1-skewed antibody response, positioning NK cells as key players in antigen processing and the instruction of adaptive humoral immunity [186].

Beyond direct killing, NK cells are potent orchestrators of the broader immune response through secretion of IFN-γ, TNF, GM-CSF and IL-6, which activate and license dendritic cells, macrophages, and T cells, while production of IL-10 and TGF-β in chronic infection settings can dampen cytotoxicity and cytokine release to limit immunopathology. They engage in bidirectional crosstalk with T cells, dendritic cells (DCs), macrophages, and neutrophils. NK-derived IFN-γ is also known to directly inhibit viral gene expression in several viral models. ln tuberculosis, HLA-DR^+^CD56^bright^ NK cells expand and provide IFN-γ that is required for optimal monocyte IL-15/IL-18 production and Th1-like CD4^+^ and CD8^+^ T cell responses against Mtb antigens. During *T. gondii* infection, NK cell derived IFN-γ contributes to early CD4^+^ and CD8^+^ T cell priming and resistance, whereas, in late chronic infection, a modified NK subset emerges that suppresses CD8^+^ T cell function and promotes exhaustion, and NK depletion at this stage restores polyfunctional CD8^+^ responses and survival, revealing the milieu effect of NK cell cytokine network. Following co-incubation with *Candida*, NK cells can prime neutrophils through there supernatant, enhancing their candidacidal activity and showing that NK-derived factors condition neutrophils for enhanced antifungal killing [174, 183, 191, 196–199, 201, 202] Moreover, activated APCs release cytokines such as IL-12, IL-15, IL-18, and IL-23 which function as critical survival and priming signals for NK cells. Dendritic cells and macrophages produce IL-12 and IL-18 In response to viruses, fungi, and vaccines, which drives robust NK-IFN-γ secretion and enhances cytotoxic granule release, thereby amplifying early antiviral and antifungal defense. In parasitic infection, IL-12 and IL-23 dependent NK responses are essential for protection against secondary *T. gondii* infection, as disruption of these cytokine pathways impairs NK-IFN-γ production and compromises control of reinfection, underscoring that APC-derived cytokines are key extrinsic signals that license full NK effector function [169, 171, 200, 203]. The Li et al. study shows that NK cells from HIV infected Individuals have reduced NKp30 expression and Impaired antifungal cytotoxicity. However, when these cells are cultured with IL-12 (often together with IL-2), NKp30 surface expression increase and their fungal killing capacity is partly restored, indicating that IL-12 can rescue NKp30 levels and functionality in NK cells In infected individuals [184].

### NK cell responses to fungal infections

Beyond the roles of NK cells and their cytotoxicity mechanisms against fungal infections discussed above, further in vitro data have confirmed that NK-92 cell lines can induce measurable hyphal damage against not only *Aspergillus* but also mucormycetes like *Rhizopus arrhizus*. Crucially, this response is modulated by accessory cells; *Candida*-dendritic cell co-cultures demonstrate that fungal pathogens shape the cytokine milieu (specifically IL-12) produced by DCs, which in turn dictates the magnitude of the NK cell response, underscoring that antifungal immunity relies on both direct receptor engagement and cytokine-driven licensing [200, 204]. In systemic *Sporothrix schenckii* infection models, splenic NK cells have been shown to expand and upregulate maturation markers like CD62L and KLRG1. Depletion of NK cells in these models results in a drastic increase in fungal burden and exacerbated systemic inflammation, confirming their non-redundant role in controlling fungal dissemination [205].

### NK cell responses to bacterial infections

While traditionally associated with antiviral defense, NK cells exert significant control over intracellular bacteria. In the case of *Pseudomonas aeruginosa*, NK cells mediate direct killing via contact- dependent degranulation [189]. However, the interaction with *Streptococcus pneumoniae* appears more complex; while NK cells directly sense the bacterium and respond with IFN-γ production and cytotoxicity, extensive screening of receptor families (Ly49, NKG2) has yet to identify a single dominant receptor-ligand pair, suggesting a multi- receptor recognition mechanism or the involvement of novel receptors [206].

The role of NK cells in tuberculosis remains a subject of intense investigation and debate. NK cells expressing HLA-DR have emerged as a specialized subset capable of bridging innate and adaptive immunity by acting as potential non-professional antigen-presenting cells. Studies by Kust et al. demonstrated that NK cells can sense mycobacterial components via TLR2, NKp44 and NKP46, leading to the expansion and cytotoxicity enhancement of an HLA-DR+ subset. These cells, when pulsed with Mtb antigens can stimulate CD4^+^ T cells to produce IFN-γ and TNF, thereby amplifying the Th1 response required for macrophage activation and control of Mtb [174]. However, the functional competency of NK cells in TB appears compartment-specific. Young et al. reported a striking dichotomy: while peripheral blood NK cells in TB patients displayed a mature, highly cytotoxic phenotype driven by T cell derived IL-2, NK cells isolated from infected lung tissue were predominantly immature and hypo-functional. This suggests that the local granulomatous environment may actively regulate NK effector function or restrict the entry of mature effectors [207]. Furthermore, the role of NK cells in TB is not universally protective; Silva et al. found no significant differences in total NK cell counts or functionality between healthy individuals and those with latent or active tuberculosis, suggesting context-dependent variability in NK cell involvement [208].

### NK cell responses to parasitic infections

NK cell efficacy in parasitic infections is highly context-dependent, often varying by the life-cycle stage of the pathogen. In malaria, NK cells are critical for the control of the pre-erythrocytic liver stage. Research indicates that hepatic NK cells in infected hosts acquire potent cytotoxicity against *Plasmodium yoelii* intracellular parasites within hepatocytes in infected mice. However, these same cells often lack direct cytotoxicity against the erythrocytic (blood) stage of the parasite [209]. Nevertheless, multiple studies show that NK cells also act at the blood stage of malaria. Human NK cells can recognize *Plasmodium falciparum*-infected erythrocytes, upregulate granzyme B, and directly lyse infected RBCs, and NK cell depletion in humanized mice leads to higher parasitemia, confirming their role in controlling erythrocytic infection. Furthermore, antibodies from *Plasmodium*-exposed individuals opsonize infected erythrocytes and elicit potent NK-mediated ADCC that selectively kills infected RBCs and suppresses blood-stage parasite growth. Crucially, repeated *Plasmodium* exposure induces the expansion of a distinct, adaptive-like NK phenotype; these cells are CD56^−^, and deficient in both the signaling adaptor FcRγ and the transcription factor PLZF, yet they express high levels of the activating receptor NKp30. Despite the loss of classical markers, this subset exhibits rapid activation kinetics and enhanced ADCC capacity against infected erythrocytes, correlating with lower parasitemia and reduced clinical risk [193, 210–214].

The influence of cytokine milieus is particularly evident in *T. gondii* infection. NK cells are indispensable for early resistance, driven by IL-12 and IL-23. However, during chronic infection, the NK compartment can become detrimental. lvanova et al. demonstrated that chronic antigen exposure drives the emergence of an NKp46^high^NKG2A^+^ subset that actively suppresses CD8^+^ T cell responses, contributing to T cell exhaustion. Blocking NKp46 or depleting this specific NK subset revitalized CD8^+^ T cell function and improved survival, highlighting a regulatory check that NK cells can place on adaptive immunity during persistence [196, 203]. On the other hand, in *Trypanosoma cruzi* infection (chagas disease), NK cells are vital for both primary control and vaccine efficacy. They localize to the epidermis, the site of vector bite, interacting with infected keratinocytes. Vaccine studies using attenuated parasites show that protection is dependent on the rapid recall of “experienced” NK cells and genetic NK cell deficiency markedly weakens the protection conferred by immunization, suggesting a memory-like component to the protection against this protozoan parasite [183, 194].

### Adaptive and “Memory-Like” NK cells

Recent studies have challenged the view of NK cells as short-lived, purely innate effectors by demonstrating that specific NK subsets can develop durable, antigen-specific “memory-like” properties, especially in the setting of CMV, HIV, and *Influenza* infection. These adaptive NK cells are frequently marked by upregulation of the activating receptor NKG2C and rely on recognition of pathogen-derived nonameric peptides presented by HLA-E, which has been mechanistically demonstrated for CMV UL40 as well as HIV and *Influenza* peptides. Modulation of NK cell receptors, such as NKG2C and other immune checkpoints, is notably significant in COVID-19 infection. However, while fully characterized, long-lasting NKG2C-defined adaptive memory NK responses are well-documented for CMV, HIV, and *Influenza*, they remain less clearly established for COVID-19 [175, 192, 215, 216].

In a concept-challenging study, Camarasa et al. showed that, in a mouse model of *Streptococcus pneumoniae* infection, NK cells develop specific and long-term memory, and that transfer of these memory NK cells confers antigen-specific protection against lethal *S. pneumoniae* challenge for at least 12 weeks, thereby further undermining the long-standing view that immunological memory is restricted to the adaptive compartment [206]. Similarly, in *Ehrlichia* infections, the depletion of NK cells prior to secondary challenge leads to reduced IFN-γ production by memory T cells, higher bacterial burden, and impaired protective recall immunity, suggesting NK cells are integral to maintenance or activation of adaptive memory [217]. However, this property is not universal; in *Toxoplasma* models, while NK cells are required for secondary control, they appear to rely on extrinsic cytokine cues (IL-12/IL-23) rather than intrinsic antigen specific memory programming [196, 203].

### Clinical applications

The potent cytotoxic capability of NK cells, combined with their safety profile, specifically the lack of HLA- matching requirements and the absence of GVHD, has led to their rapid development as adoptive immunotherapies for refractory infectious diseases.

In the context of the COVID-19 pandemic, NK cell therapy was evaluated as a novel intervention. A phase I RELEASE trial assessed the infusion of ex vivo expanded NK cells from convalescent donors into patients with moderate COVID-19 pneumonia. The results showed the therapy was safe, with no dose-limiting toxicities or cytokine release syndrome (CRS), and was associated with rapid viral clearance, resolution of inflammation, and survival in all treated patients [218]. Parallel studies using of-the-shelf, umbilical cord blood- derived NK cells (DVX201) in hospitalized COVID-19 patients similarly demonstrated a strong safety profile and stabilization of inflammatory biomarkers, validating the feasibility of allogeneic NK products in acute viral settings [219]. Beyond COVID-19, NK cell infusions have shown efficacy in controlling viral reactivation in immunocompromised hosts. In haplo-HSCT recipients involving pediatric patients with Wiskott-Aldrich syndrome, the administration of donor-derived, ex vivo-expanded NK cells significantly declined CMV DNA levels, improved of CMV pneumonia and retinitis compared to historical controls, without GVHD or major infusion related toxicity. Another clinical study including refractory CMV further support this with a significantly lower incidence of CMV and refractory CMV infection, shorter CMV DNAemia, and a trend toward lower peak viral load [220, 221].

Furthermore, genetic engineering is enhancing NK cell specificity. CAR-NK cells, initially developed for cancer, are now being adapted for infection. A pioneering study demonstrated that NK-92 cells engineered with a scFvk3-1 receptor targeting fungal cell wall mannan exhibited markedly increased degranulation, and IFN-γ release, and successfully reduced fungal burden in mice infected with *Candida albicans*. This proof-of-concept establishes that NK cells can be redirected against other pathogens through synthetic biology [222].

## Cytokine-induced killer (CIK) cells

### Ontogeny, definition, and cellular biology

Cytokine-induced killer (CIK) cells were originally developed in the seminal work of Schmidt-Wolf and colleagues as a heterogeneous population of ex vivo IFN-γ/anti-CD3/IL-2-expanded cytotoxic lymphocytes with potent antitumor activity in a SCID mouse/human lymphoma model and proposed for adoptive immunotherapy. Over the subsequent three decades, CIK therapy has been evaluated in numerous clinical trials and is now approved in several countries, including Germany, particularly in the setting of solid tumors such as lung cancer [30, 223]. Regarding the extent of oncology clinical experience, the 2020 International Registry on CIK Cells report included 106 studies comprising 10,225 participants, of whom 4,889 received CIK cells alone or in combination with other therapies with improved progression-free survival and overall survival [224]. These cells merge functional attributes of both T cells and NK cells, providing a rationale for investigating their activity against infections in addition to malignancies.

Derived from peripheral blood mononuclear cells (PBMCs), lymphoid-derived sources, or umbilical cord blood, CIK cells are generated ex vivo through stimulation with a defined cytokine cocktail, IFN-γ, IL-1β, IL-2, and anti-CD3 antibody. In recent clinical protocols, this cocktail is frequently supplemented with IL-15 to maximize viability and cytotoxicity [224]. This protocol achieves a mixed T cell population that acquires NK like features, such as CD56 expression and the capacity for both MHC-dependent and MHC-independent cytotoxicity [225]. CIK cultures comprise CD3^−^CD56^+^, CD3^+^CD56^+^, and CD3^+^CD56^−^ subsets, with the CD3^+^CD56^+^ fraction, enriched in CD8 late effectors, exhibiting dominant cytotoxicity against tumor/AML targets [226, 227]. The relative proportions of these subsets vary among preparations. In our recent in-vitro malaria study, the expanded CIK population encompassed roughly 73% CD3^+^CD56^−^T cells and 26% CD3^+^CD56^+^ NK-like T cells at day 14, demonstrating the heterogeneous composition of the preparation investigated against *P. falciparum* [228]. In a clinical study of autologous CIK cells administered to patients with breast cancer, the final products contained a median of 87.9% CD3^+^ T cells (range 75.9–93.4%), including a median of 20.3% CD3^+^CD56^+^ cells, and 7.0% CD3^−^CD56^+^ NK cells (range 4.5–11.1%). These median percentages correspond to an approximate T cell/NK-cell ratio of 12.6:1, calculated from the reported medians rather than individual product ratios [229]. Although αβ T cells predominate, γδT cells are not necessarily absent. In an independent experimental study, TCRαβ + and TCRγδ + cells accounted for 83.7 ± 7.9% and 11.9 ± 4.1%, respectively, of the CD3^+^CD56^+^ subset after expansion, indicating that this fraction can contain both T cell lineages [230].

Evidence further indicates that DC activated CIK cells amplify their cytotoxicity, promote proliferation, and may extend patient survival. Separately, CAR engineered CIK cells deliver elevated expression of granulysin, perforin, and IFN-γ relative to CAR-T cells, yielding more focused and prolonged cytolytic performance [231, 232].

### Mechanisms of action: The dual-signaling paradigm

The recognition mechanisms of CIK cells have been characterized predominantly against malignant targets, where TCR-mediated recognition and NK-like cytotoxicity through activating receptors, including NKG2D and DNAM-1, contribute to their dual functionality [233]. The capacity of CIK cells to combine TCR-mediated recognition with non-MHC-restricted cytotoxicity provides a rationale for investigating their activity against infections, although the contribution of individual receptors depends on the experimental setting. Conventional T cells rely on the recognition of peptide antigens presented by MHC molecules. However, viruses and tumors frequently downregulate MHC expression to escape T cell surveillance. CIK cells retain TCR-mediated cytotoxicity, while NKG2D contributes to their non-MHC-restricted killing of malignant targets. MICA/B and ULBPs are host-derived stress-associated ligands rather than pathogen-associated molecular patterns. Upon ligation, NKG2D on CIK cells signals through the adaptor molecules resulting in granule polarization and degranulation independent of TCR engagement. This allows CIK cells to effectively lyse targets that have lost MHC expression. Notably, NKG2D ligands have been observed during infections such as, CMV, HBV, EBV, adenovirus, measles, RSV, and fungal or bacterial pathogens including *Candida*, *Cryptococcus*, *Aspergillus*, and *Pseudomonas aeruginosa*. Furthermore, MICA/B and Rae-1 expression increases in infections involving *Escherichia coli*, *Mycobacterium tuberculosis*, *Influenza*, and HIV via ULBP upregulation, providing potential CIK cell targets [108, 234–237]. Infection-associated changes in host NKG2D ligands provide a rationale for investigating this pathway in CIK cells [234, 237]. Separately, direct NKG2D-mediated fungal recognition and killing have been demonstrated in NK cells [236]. However, these findings do not establish the corresponding mechanism in CIK cells. In the CMV peptide-loaded model examined by Pievani et al., NKG2D blockade did not inhibit antigen-specific CIK cytotoxicity [233].

Following activation, CIK cells deployed a multifaceted cytotoxic arsenal mirroring CTLs, γδT, and NK cells, including granule-mediated cytotoxicity characterized by robust expression of granzyme B, granulysin, and perforin. Beyond granule exocytosis, CIK cells can engage death receptor pathways, including Fas-L- and TRAIL-dependent apoptosis, adding a receptor-mediated component to their killing profile. In some CIK products, CD16 expression enables the potential for ADCC [225, 232, 238–243].

While directly inducing target apoptosis, CIK cells secrete a diverse array of cytokines, including IL-2, IFN-γ, TNF, IL-6, GM-CSF, IL-10, L-13, and chemokines (e.g., IL-8, MIP-1a/β, RANTES), that potently augment innate and adaptive responses, recruit effector cells, promote DC maturation, prevent the immunosuppressive remodeling of tissue microenvironment, and enhance their own proliferation/cytotoxicity. Transcriptomic analyses reveal distinct profiles shaped by culture conditions, with IL-2 driving IFN-γ /cytokine signaling and IL-15 favoring adhesion/proliferation pathways essential for cytolytic capacity. Exogenous IL-12 synergizes by shortening enrichment time, boosting IFN-γ/IL-2 output, and achieving up to 75% tumor remission rates, while IL-15 further optimizes expansion and function in clinical-grade protocols [224, 239, 244–249].

### Safety, manufacturing, and translational considerations

Although infection-specific head-to-head comparisons with CTLs, NK cells, or γδT cells have not yet been performed, several biological and manufacturing characteristics provide a rationale for investigating CIK cells in infectious diseases, but their comparative advantages over other effector populations remain to be established. In the broader landscape of cellular immunotherapies, CIK cells offer a distinct combination of T cell expansion and antigen-specific functions with NK-like, MHC-unrestricted cytotoxicity. Unlike CAR-T cells, CIK cells do not require obligatory genetic receptor engineering or viral vectors and can be manufactured from PBMCs using defined cytokines and anti-CD3 within GMP-compatible procedures [32, 250]. Their production therefore avoids the vector-specific testing associated with gene-transfer vectors. However, product-specific release testing remains necessary, and these differences do not establish lower overall manufacturing costs or greater feasibility in infectious diseases. Cappuzzello et al. estimated the cost of consumables and reagents for CIK production at less than €5,000 per batch, excluding personnel, quality control, GMP facility-related costs, and plant depreciation; the proposed overall cost advantage over CAR-T cells remained an estimate rather than a direct economic comparison and does not establish the total cost of a treatment course or cost-effectiveness relative to standard antimicrobial treatment or alternative cellular therapies [250]. GMP-compliant CIK production nevertheless requires qualified GMP cell processing facilities, trained personnel, validated procedures, and product-specific quality-control, and release testing which needs to be accounted [251, 252]. A comparative review of clinical studies in NSCLC further concluded that CIK cell therapy showed relatively stronger antitumor efficacy and fewer adverse effects than CAR-T cell approaches, although additional clinical studies were considered necessary to confirm these findings [253]. Though this evidence is derived from oncology and cannot be extrapolated directly to infectious diseases, it reinforces the translational rationale for investigating CIK cells as a cellular platform with potentially lower manufacturing complexity and a favorable tolerability profile.

Compared with conventional pathogen-specific CTLs, standard CIK generation does not inherently require prior identification of immunodominant pathogen-derived epitopes, antigen-specific cell selection, or restriction to a defined peptide-HLA combination [254]. CIK cells nevertheless retain TCR-mediated, HLA-restricted recognition, while the CD3^+^CD56^+^ fraction acquires additional TCR-independent cytotoxicity mediated by receptors including NKG2D, DNAM-1, NKp30, and CD16 [230, 233]. This dual functionality combines conventional TCR mediated antigen specific activity, which is absent from NK cells, with NK-like recognition of stressed or HLA-modified targets. Compared with γδT cells, CIK cultures retain conventional αβ TCR-mediated responses and antigen-specific T cell memory within the same heterogeneous product that contains MHC-unrestricted effectors. Direct comparisons nevertheless indicate that proliferative and cytotoxic performance depends on the expansion protocol and target investigated. NK or γδT cells may display greater activity against selected tumor targets, whereas CIK cells have demonstrated recognition of both MHC class I-positive and -negative tumor targets and stronger immediate cytotoxicity than conventionally expanded polyclonal T cells in other tumor models [238, 255, 256]. These findings define comparative biological properties but do not establish superior efficacy against infectious diseases.

The flexibility of this platform is further demonstrated by pathogen-directed CIK cultures. CMV-pp65 stimulated and EBV peptide-directed CIK cells, with targeted as well as broader cytotoxicity properties and low adverse effects, indicate that pathogen-specific TCR-mediated activity can be added to an existing MHC-unrestricted cytotoxic background without requiring a different cellular platform. This combination may be particularly relevant after HSCT, where viral reactivation, impaired antigen-specific immunity, and malignant relapse can coexist [257, 258].

CIK cells also undergo robust ex vivo expansion over approximately three weeks, with the initially rare CD3^+^CD56^+^ fraction capable of approximately 1000-fold or greater expansion, enabling the generation of clinically relevant cell numbers and the preparation of multiple doses. Clinical manufacturing studies have also demonstrated the feasibility of cryopreserving CIK doses, with GMP-manufactured products retaining viability, identity, and potency within six months of storage under the conditions examined [251, 252]. Advance preparation could therefore separate the expansion period from the interval between treatment indication and administration, provided that a suitable product has already been manufactured and released. This provides a logistical rationale for investigating banked CIK products in acute as well as chronic infections, although storage alone does not establish suitability for unrestricted third-party use or clinical benefit in acute infection.

CIK cells display reduced alloreactive potential compared with conventional donor T cell products, although the risk of GVHD is reduced rather than absent. Their cytotoxicity is mediated substantially through activating receptors such as NKG2D rather than exclusively through TCR recognition of recipient alloantigens, supporting their cautious use across HLA barriers [231, 259]. Beyond GVHD, reported adverse effects of conventional CIK cell administration include transient fever, chills, fatigue, and headache, generally of low grade [260]. A recent multicenter study reported 169 infusions of IL-15 activated CIK cells in 56 post-HCT treatment cases, including donor-recipient pairs classified as ≥ 9/10 HLA-matched or ≤ 8/10 HLA-mismatched according to the study definitions, and reported predominantly mild IL15-CIK-associated adverse events, with grade 1–2 and grade 3 acute GVHD occurring in 27% and 4% of cases overall, respectively. The two grade 3 acute GVHD cases occurred in one HLA-matched recipient treated preemptively and one HLA-mismatched recipient receiving consolidation therapy; both were manageable and reversible with standard treatment. Limited chronic GVHD developed in two cases in each donor group, and no grade 4 acute GVHD was observed. One grade 1 CRS event was reported in the overall cohort [261]. These findings concern donor-derived CIK cells administered after HCT with characterized donor-recipient HLA compatibility and should not be interpreted as evidence supporting administration without HLA typing. Safety findings should nevertheless be distinguished according to the cellular product. In a study of genetically modified CAR-CIK-CD19 cells, grade ≤ 2 CRS occurred in 15 of 36 patients, grade 2 immune effector cell-associated neurotoxicity syndrome (ICANS) in one patient, and late onset grade 3 peripheral neurotoxicity in two patients; these findings should not be directly extrapolated to conventional, non-CAR-engineered CIK cells [262]. Limited reporting cannot, however, exclude rare or delayed events. These observations describe safety in the populations and treatment settings examined; they do not establish a lower risk of adverse events than alternative cellular therapies in patients with infectious diseases.

In allogeneic HSCT recipients, available evidence does not establish a routine requirement for preemptive reduction, withdrawal or intensification of immunosuppression solely because CIK cells are administered. Clinical approaches have differed: Introna et al. administered donor-derived CIK cells after complete withdrawal of cyclosporine and corticosteroids, whereas Narayan et al. evaluated early post-transplant CIK consolidation within a cyclosporine/mycophenolate-based GVHD prophylaxis regimen [32, 263]. Neither study compared immunosuppression strategies, and both concerned hematologic malignancies rather than treatment of infection. Consequently, these findings do not define an optimal regimen for infection-directed CIK therapy. Prospective studies are needed to determine how concomitant immunosuppression influences CIK persistence, antimicrobial activity and GVHD risk.

Phenotypically, mature CD3^+^CD56^+^ CIK cells predominantly display an effector memory profile, typically defined by CD45RO expression and the loss of lymph node homing markers (CD62L, CCR7), together with low levels of CD27 and CD28. In contrast, the accompanying CD3^+^CD56^−^ subset retains comparatively earlier memory characteristics with higher CD62L^+^ level. Consequently, CIK cultures expanded via standard IFN-γ/anti-CD3/IL-2 protocols maintain substantial heterogeneity, encompassing CD45RA^+^CD62L^+^ naïve and CD45RO^+^CD62L^+^ central memory cells alongside the dominant CD45RO^+^CD62L^−^ effector memory fraction. This diverse cellular composition may influence function: the large pool of CD3^+^CD56^+^ effectors ensures potent immediate cytotoxicity, whereas the reservoir of naïve and central memory cells may support in vivo persistence and proliferative potential [242, 264–267].

Although evidence concerning CIK cell trafficking to the central nervous system (CNS) remains limited, in a preclinical glioblastoma study, intravenously administered CIK cells infiltrated intracranial tumors in mice, demonstrating access to brain tumors under the conditions examined [268]. Clinical experience also includes local administration of autologous CIK cells into glioma resection cavities; however, this approach bypasses the blood-brain barrier and does not establish trafficking from the circulation [269]. Evidence of cerebrospinal fluid (CSF) detection has also been reported for genetically engineered CIK products. Lussana et al. detected donor-derived CAR-CIK-CD19 cells in the cerebrospinal fluid of a patient with a history of CNS leukemic involvement [262]. This observation documents CSF access by a CAR-engineered CIK product, but does not establish equivalent trafficking of conventional CIK cells or activity against CNS infections. Direct evidence documenting recovery of systemically infused conventional CIK cells from human CSF remains lacking in the literature reviewed.

Moreover, CIK cells exhibit a variable checkpoint profile following ex vivo expansion, with generally low expression of PD-1, CTLA-4, and inhibitory KIRs, although LAG-3, TIM-3, PD-L1, and CEACAM-1 vary according to the cellular source, culture stage, and expansion protocol [270, 271]. CIK cells remain susceptible to checkpoint-mediated suppression, and blockade of PD-1, KIR, LAG-3, or TIM-3 can increase their cytotoxicity, restore NKG2D expression, and enhance cytokine secretion [270, 272]. The feasibility of combining CIK cells with PD-1 blockade has also been investigated in advanced non-small-cell lung cancer [223, 273, 274]. Although these findings originate from oncology, they indicate that CIK cell activity remains amenable to checkpoint modulation, which may become relevant in chronic infections characterized by persistent antigen exposure and cytotoxic lymphocyte dysfunction.

Taken together, the expansion capacity, antigen-specific and non-MHC-restricted cytotoxic functions, and feasibility of repeated administration and cryopreservation described in selected CIK studies provide a rationale for investigation in infectious diseases. The relative merits of CIK cells, CTLs, NK cells and γδT cells require comparison within defined infectious settings. Whether the combined CIK profile translates into superior antimicrobial efficacy, clinical benefit, or cost effectiveness relative to standard antimicrobial treatment or alternative cellular therapies remains to be established in infections specific comparative studies.

Comparative evaluation should distinguish experimental activity from clinical benefit. Laboratory comparisons with CTLs, NK cells or γδT cells should use the same infected targets and comparable assay conditions to assess antimicrobial activity and injury to uninfected cells. Clinical comparisons should account for the pathogen, disease severity, immune status and concomitant antimicrobial treatment, with prespecified microbiological and clinical endpoints and consistent assessment of adverse events during follow-up. Feasibility should be assessed through manufacturing success, product-release requirements and the interval from treatment indication to infusion. Economic comparisons should include the full treatment course, including cell collection, personnel, facilities, quality control, storage, transport, repeated administration and adverse-event management. Claims of greater accessibility would additionally require evidence that suitable products can be delivered within the necessary clinical timeframe in the intended healthcare setting.

### Therapeutic applications in infectious settings

Despite extensive oncology research, mechanistic exploration of CIK cells in infectious disease contexts remains limited, largely confined to studies involving oncogenic viruses and specific infectious setting. Consequently, the pathways by which CIK cells engage pathogens remain under characterized. The direct infection-specific studies of CIK cells are summarized in Table 1, including their settings, study designs, sample sizes, comparators, concomitant or preceding treatments, main outcomes and key limitations. Our group recently demonstrated that CIK cells significantly reduce *P. falciparum* parasitemia in vitro; however, this study did not establish the receptor-ligand interaction responsible for recognition of infected erythrocytes. In these co-cultures, parasite control was predominantly secretion dependent, as shown in the transwell system, with CIK cells releasing granzyme B, perforin, and particularly granulysin at significantly elevated levels, mirroring potentially the granulysin-mediated delivery of granzyme B into infected erythrocytes described for other cytotoxic lymphocytes in malaria; lower granulysin secretion was associated with ineffective antiparasitic activity in the donors examined. The cytotoxicity mechanism and antiparasitic activity were retained in the transwell system without needing cell-cell contact. However, when CIK cells were in proximity to infected red blood cells, an additional 10% increase in cytotoxicity was observed. This suggests that while direct contact is not necessary, close interaction between CIK cells and infected red blood cells can slightly enhance the inhibition of parasitemia. Furthermore, selective recognition of infected over uninfected erythrocytes was not established [228]. Infection-associated membrane lipid changes, particularly cholesterol composition, may contribute to differential susceptibility to granulysin, as suggested by independent work [275]. This finding is consistent with the granulysin dependent activity observed in our CIK co-culture, although the contribution of membrane cholesterol to that activity was not directly tested.

**Table 1:** Direct infection-specific studies of cytokine-induced killer (CIK) cells

Study	Setting	Study type	CIK product	Sample size	Comparator	Concomitant / preceding treatment	Main outcome(s) assessed	Key limitations
A. Preclinical studies (in vitro)								
Petvises 2007 [276]	EBV, post-HSCT	In vitro cytotoxicity against autologous EBV-LCL	Conventional CIK	6 patients (3 for subset comparisons)	Autologous EBV-LCL versus K562	None (ex vivo); donors on full-dose immunosuppression	Lysis of autologous EBV-LCL and IFN-γ release	Virus-transformed, not infected targets; lysis of K562 was numerically higher than lysis of autologous EBV-LCL in the subset comparison (*n* = 3); no clinical endpoint
Pievani 2011 [233]	CMV	In vitro receptor-blocking cytotoxicity	Conventional CIK; CMV-specific cells tetramer-selected after expansion	5 donors; 3 experiments	CMV- versus EBV-peptide-loaded and unloaded autologous blasts; K562	None (ex vivo)	Antigen-specific lysis of CMV-loaded targets by tetramer-selected CMV-specific CIK cells; blockade of HLA class I, LFA-1 and DNAM-1 reduced killing, NKG2D and NKp30 did not	Peptide-loaded targets, not infected cells; blocking experiments identify contributions to cytotoxicity rather than recognition of a CMV ligand
Pfirrmann 2015 [258]	CMV and leukemia; in vitro	In vitro cytotoxicity and alloreactivity	CIK pulsed with CMV pp65 protein during expansion	18 donors screened, 13 responders; 3 for fibroblast assay	Conventional (non-pulsed) CIK in parallel; mock- versus CMV-infected fibroblasts	None (ex vivo)	Higher lysis of pp65-loaded targets; killing of CMV-infected but not mock-infected fibroblasts; leukemia lysis retained	Conventional CIK cells also killed CMV-infected fibroblasts, so the contribution of pp65-specific recognition to infected-cell killing was not established; receptor blocking was performed only against peptide-loaded T2 cells, not against infected fibroblasts (*n* = 3)
Pfeffermann 2018 [259] Laboratory	EBV	In vitro expansion, phenotyping and cytotoxicity	CIK pulsed twice with an EBV consensus peptide pool	12 donors (7 for cytotoxicity)	Non-pulsed CIK; peptide-loaded versus unloaded targets	None (ex vivo)	Selective expansion of EBV-specific effectors and higher killing of peptide-loaded targets	Peptide-loaded targets rather than infected cells; the effect required antigen pulsing during manufacture; assay-specific sample sizes range from 4 to 12 donors
Luah 2019 [277]	CMV	GMP-compliant manufacturing and potency study	CMV peptide-stimulated, CD137-selected CIK	7 blood samples	CD137-positive versus negative and unsorted cultures; conventional CMV-CTL	None (ex vivo); donors had iron overload or polycythemia	High-level expansion with CMV-specific lysis and minimal killing of non-loaded targets; specificity retained after cryopreservation	No patient administration; non-standard donor source; cryopreservation reduced potency, tested in 2 samples
Vaseq 2026 [228]	P. falciparum (malaria)	In vitro co-culture and transwell assay	Conventional CIK	3–6 donors per assay	Infected erythrocytes without CIK cells; transwell versus direct contact	None; antimalarial drugs tested in separate experiments	Significant reduction in parasitemia, retained in transwell culture; granulysin secretion higher in effective donors	Recognition receptor not identified; selective recognition of infected erythrocytes not established; donor-dependent activity
B. Clinical studies								
Shi 2009 [278]	Chronic hepatitis B	Prospective single-arm; 24 weeks	Autologous conventional CIK	14	Pretreatment values; no control group	None; antiviral therapy was an exclusion criterion	Virological response in 7 of 14; HBeAg loss in 4 of 7 and seroconversion in 2 of 7; dendritic cell recovery in responders only	No control group; the responder split is post hoc and confounded by higher CD3^+^CD56^+^ content in responders’ products
Shi 2009 [279]	Chronic hepatitis B	Prospective single-arm; 48 weeks	Autologous conventional CIK	21	Pretreatment values; no control group	None during the study; 6 patients had prior antiviral therapy, discontinued over 6 months earlier	Virological response 23.8% overall: 0% versus 57.1% for baseline ALT of 40 U/L or less versus above (*p* < 0.05)	No control group; the virological response was confined to patients with baseline ALT above the normal limit of 40 U/L, with no responses below it; 6 patients had pre-existing antiviral drug resistance
Ma 2013 [280]	Chronic hepatitis B	Controlled; allocation method not described	Autologous DC-CIK (HBsAg-activated DC co-cultured with CIK)	33 versus 27	Symptomatic treatment alone	The control group received symptomatic treatment only, without antiviral or immunosuppressive therapy	HBV DNA reduction of at least 2 log in 21 of 33 (63.6%) versus 4 of 27 (14.8%); *p* < 0.001	No randomization described and arms differ in age; the DC-CIK combination prevents attribution to CIK cells
Cui 2014 [281]	HCV within an HCC cohort	Open-label controlled; patients self-selected treatment	Combined NK, γδ T and CIK product	30 versus 32 (3 HCV per arm)	RFA alone	RFA; no antiviral therapy in the HCV patients	Longer progression-free survival with RFA plus cell therapy; HCV titers fell in 2 of 3 treated patients and rose in all 3 controls	Allocation by patient self-selection; 3 patients per arm, HCV subgroup described descriptively, with no p-value reported; combined cellular product
Qian 2016 [282]	HCV within an HCC cohort	Retrospective controlled	Combined NK, γδ T and CIK product	31 versus 73 (28 versus 68 analyzed)	Conventional therapy alone	Conventional anticancer treatment; interferon and ribavirin excluded	No viral increase in the treated group (0 of 28) versus 17 of 68 controls; decreased or stable in 100% versus 75% (*p* = 0.014); median PFS 16 versus 10 months	Retrospective with probable selection bias; combined cellular product
Feng 2017 [283]	Chronic hepatitis B	Uncontrolled single-arm DC-CIK study	Autologous DC-CIK	14	Pretreatment values; no control group	GM-CSF mobilization before collection	HBsAg reduced (*p* = 0.041); HBV DNA, HBeAg and liver function measures showed no statistically significant change	No control group; HBsAg was the only significant endpoint; no safety data reported
Pfeffermann 2018 [259] Clinical case	EBV-associated lymphoma, post-HSCT	Single compassionate-use case	EBV-peptide-pulsed CIK	1	None	Rituximab, EBV-specific T cells and conventional CIK given earlier, 20 and 10 days before the final infusion	Remission within 7 days with viral clearance, sustained at 2.5 years	Single patient; preceding therapies prevent attribution; chronic GVHD at 6 months, with a relationship to treatment not excluded
Xu 2018 [284]	MDR pulmonary tuberculosis	Single-patient case report	Autologous conventional CIK	1	Persistent positive cultures after 4 months of the same regimen alone	Six-drug second-line regimen throughout (moxifloxacin, amikacin, pyrazinamide, amoxicillin–clavulanic acid, para-aminosalicylic acid, and protionamide) continued for a further year	Smear negative at 2 months and culture negative at 3 months; no relapse at 2 years	Single case; the authors state that the continued regimen prevents attributing relapse-free survival to CIK cells
Tang 2022 [285]	MDR pulmonary tuberculosis	Retrospective controlled	Autologous conventional CIK	9 versus 9	Chemotherapy alone	WHO multidrug regimen; recombinant IL-2 included in the infusion solution	At three months smear negativity 8 of 9 versus 2 of 9 (*p* = 0.015); culture negativity 9 of 9 versus 5 of 9 (*p* = 0.082, not significant)	Retrospective with 9 per arm; significant baseline imbalance; the culture difference did not reach significance

Inclusion criteria: studies in which a CIK or CIK-containing cellular product was tested against an infectious agent, against infected or virus-transformed cells, or in patients with an active infection. Mechanistic studies conducted exclusively in oncology models, and infection-related findings in other cytotoxic lymphocyte populations, are summarized in Table 2. Pfeffermann et al. [258] appears in both sections because its laboratory and clinical components differ in design. The studies differ in whether the CIK product itself was exposed to pathogen antigen. For controlled clinical studies, numbers are presented as intervention versus control. Laboratory sample sizes refer to donors or independent experiments, as specified

Abbreviations: ALT, alanine aminotransferase; CIK, cytokine-induced killer; CMV, cytomegalovirus; DC, dendritic cell; EBV, Epstein-Barr virus; GM-CSF, granulocyte-macrophage colony-stimulating factor; GVHD, graft-versus-host disease; HBV, hepatitis B virus; HCC, hepatocellular carcinoma; HCV, hepatitis C virus; HSCT, hematopoietic stem cell transplantation; LCL, lymphoblastoid cell line; MDR, multidrug-resistant; NK, natural killer; PBMNC, peripheral blood mononuclear cells; PFS, progression-free survival; RFA, radiofrequency ablation

Earlier work by Petvises et al. demonstrated that CIK cells generated from pediatric patients after stem cell transplantation exhibited cytotoxicity against autologous EBV-transformed lymphoblastoid cell lines. These findings provided preclinical evidence of CIK activity against EBV-transformed targets, although clinical efficacy and control of lytic EBV infection were not evaluated [286]. Pfeffermann et al. pioneered EBV-specific CIK cells, generated by pulsing with EBNA1/LMP2A peptides, achieving 10-fold selective expansion of NKG2D-high CD3^+^CD8^+^ effectors that doubled cytotoxicity against EBV-peptide-loaded targets while retaining broad K562 lysis. In a compassionate-use case of rituximab-resistant, post-HSCT CD20^−^ diffuse large B-cell lymphoma, a single infusion of EBV-specific CIK cells was followed by complete clinical remission within 7 days and viral clearance, without CRS or acute toxicity. EBNA1-specific CD4^+^ T from infused cells remained detectable in peripheral blood for up to 32 days by flow cytometry using an HLA-DRB1*04:01/EBNA1 tetramer. This approach monitored a particular antigen-specific T cell population rather than uniquely identifying the entire heterogeneous CIK product [258]. Pievani et al. demonstrated that CMV-specific CD3^+^CD56^+^ CIK cells retained antigen-specific cytotoxicity against autologous targets loaded with CMV pp65 peptide. Blocking HLA class I, LFA-1 or DNAM-1 reduced this activity, whereas NKG2D and NKp30 blockade did not inhibit CMV peptide-specific killing. The DNAM-1 and LFA-1 blocking results support their contribution to cytotoxicity in this antigen-loaded model, rather than recognition of a CMV-derived ligand by these receptors. These findings demonstrate functional recognition of CMV antigen in a peptide-loaded target model, although activity against naturally infected cells and clinical control of CMV infection were not examined in this study [233]. Similarly, Pfirrmann et al. developed CMV-pp65 peptide-pulsed CIK cells from seropositive donors that co-expanded CMV specific effectors with high NKG2D, delivering superior lysis of pp65-loaded T2 cells/CMV infected fibroblasts while retaining leukemia killing and exhibiting low alloreactivity akin to conventional CIK. These cells mounted robust IFN-γ responses upon restimulation (peaking early post-exposure) alongside degranulation/chemokine release (e.g., MIP-1a correlates), enabling dual CMV/leukemia targeting without toxicity, a promising strategy for post-HSCT complications [257]. Luah et al. further developed a GMP-compliant approach for generating CMV-specific CIK cells through stimulation with pp65 and IE-1 peptides and CD137 co-stimulation. The resulting cultures predominantly comprised late effector memory CD8 + T cells, exhibited CMV-directed cytotoxicity, and retained functional activity after cryopreservation. This study supported the feasibility of manufacturing an antigen-directed CIK product, but did not evaluate clinical efficacy against CMV disease [276]. These pathogen-directed cultures provide direct evidence of infection-related CIK activity, but the contribution of NKG2D to infected targets recognition and subsequent cytotoxicity requires a direct experimental confirmation.

In a clinical study of autologous CIK transfusion for chronic HBV (*n* = 14), 7/14 patients achieved sustained HBV DNA reduction with HBeAg loss/seroconversion, without serious toxicity or major ALT fluctuations, establishing excellent safety. Mechanistically, virological responders had CIK products enriched in CD3^+^CD56^+^ effectors, and therapy selectively restored myeloid/plasmacytoid DC frequency and cytokine production, changes correlating directly with viral suppression rather than ALT normalization [277]. Another study confirmed robust proliferation and functional activity of chronic HBV patient derived CIK cells, including increased CD3^+^CD8^+^ and CD3^+^CD56^+^ populations, upregulated CD107a, and downregulated PD-1. These changes were associated with reductions in HBV DNA load and ALT improvement among treated patients, with greater improvements observed in individuals whose baseline ALT exceeded 40 U/L, while safety laboratory results remained stable across hepatobiliary and metabolic parameters. This finding complemented by responder enriched CD3^+^CD56^+^ subsets in related work, underscoring CIK cells therapeutic potential in HBV [278]. Clinical studies have also evaluated CIK cells in combination with HBsAg-activated dendritic cells. Ma et al. reported a reduction in HBV DNA of at least 2 log in 21 of 33 patients receiving autologous HBsAg-activated DC-CIK cells, compared with four patients in the control group, corresponding to response rates of 63.6% and 14.8%, respectively [279]. However, the findings have not been consistent across studies. In an uncontrolled study of 14 patients with chronic HBV infection, Feng et al. observed a reduction in HBsAg following autologous DC-CIK treatment, whereas HBV DNA, HBeAg, and liver function parameters did not change significantly [280]. These observations indicate variable responses to DC-CIK therapy and do not establish the independent contribution of CIK cells or the mechanisms responsible for viral suppression.

Combined cellular immunotherapy incorporating NK, γδT, and CIK cells has been evaluated in patients with HCV-associated hepatocellular carcinoma. Cui et al. reported decreased HCV viral load in two of three HCV-positive patients receiving this combined treatment following radiofrequency ablation without antiviral therapy [283]. Similarly, Qian et al. reported decreased or stable HCV RNA levels in patients receiving the three cell populations alongside conventional cancer treatment [281]. Neither study isolated the contribution of CIK cells; therefore, these observations do not establish a CIK-specific recognition pathway or an independent antiviral effect against HCV and should be distinguished from receptor blocking evidence obtained in defined target cell models.

Beyond viral infections, an earlier case report described sputum culture conversion and absence of relapse during a two-year follow-up in a patient with multidrug-resistant pulmonary tuberculosis who received autologous CIK cells alongside second-line anti-tuberculosis treatment. The concurrent antimicrobial treatment and single-patient design precluded attribution of the response to CIK cells alone [282]. In a retrospective clinical study, Tang et al. demonstrated that nine patients undergoing conventional MDR-TB chemotherapy combined with a three-month regimen of repeated infusions of ex vivo expanded autologous CIK cells experienced significantly improved clinical outcomes. These included faster sputum smear and culture conversion, enhanced radiographic lesion absorption, and more rapid symptom relief compared to chemotherapy alone. The therapy was well tolerated, with no major safety concerns, and was linked to increased lymphocyte proportions and decreased soluble IL-2 receptor levels. Overall, the findings indicate that adjunctive CIK immunotherapy may accelerate bacterial clearance and promote quicker clinical recovery in MDR-TB patients. In this study, repeated infusions used autologous CIK cells, with each patient remaining the cellular source throughout treatment. The observed improvements therefore support potential clinical activity during repeated administration alongside chemotherapy, however, additional benefit of repeated versus single infusion requires additional exploration [284]. In oncology, a phase I study of donor-derived CIK cells after allogeneic transplantation, 11 patients with relapsed hematological malignancies received a median of two infusions, with three achieving complete responses, one showing hematologic improvement, and one maintaining stable disease. However, the uncontrolled study design and preceding salvage treatments limit attribution of these outcomes to CIK cells or to repeated administration specifically [32]. Continued use of the original stem-cell donor for successive CIK preparations has been described, supporting the feasibility of repeated treatment from the same donor, although these oncology findings do not establish efficacy in infectious diseases [252].

These studies show CIK cell investigation in selected viral, bacterial, and parasitic infections but do not establish efficacy throughout the corresponding pathogen categories. Although shared effector mechanisms provide a coherent biological rationale, differences in cellular preparation, experimental design, concomitant treatment, and outcome assessment limit generalization across infectious settings.

### Emerging applications: Targeting parasitic, bacterial, and fungal pathogens

Beyond the selected viral, bacterial, and parasitic settings investigated to dates, CIK cells warrant investigation against additional pathogens, particularly where effective standard treatment is unavailable, poorly tolerated, or compromised by resistance. Potential settings include antibiotic-resistant bacterial or mycobacterial infections, refractory disease associated with JC, BK, HHV-8, influenza, or SARS-CoV-2, and invasive fungal infections such as mucormycosis and aspergillosis. Fungal applications remain a research hypothesis in CIK cells, as the evidence discussed arises from other cytotoxic lymphocytes rather than direct CIK cells investigations. The efficacy and safety of CIK cells require further evaluation in each proposed infectious setting, particularly in immunosuppressed patients.

**Fig. 3:**
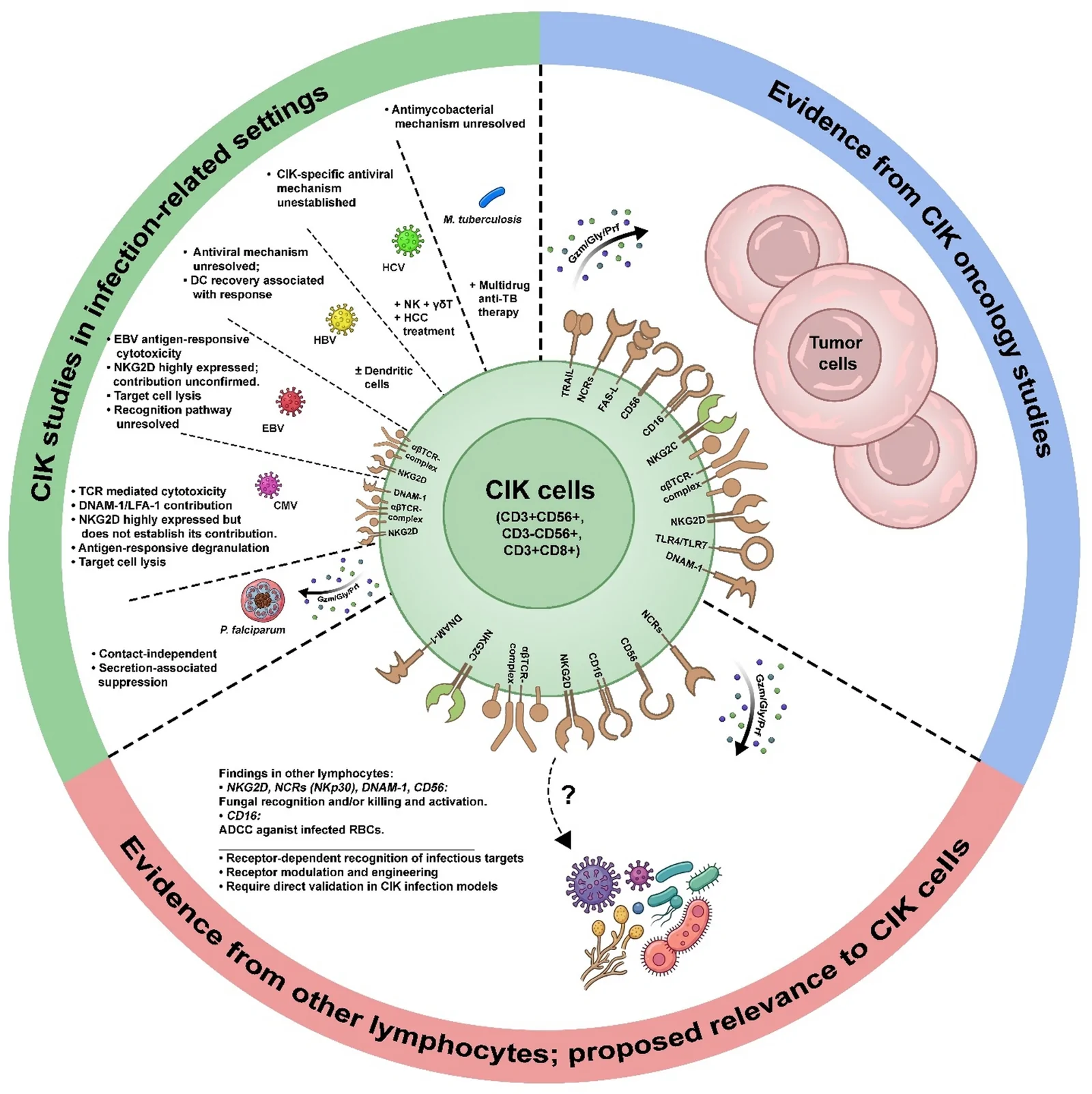
Experimental evidence and proposed mechanisms of CIK activity in infection-related settings. The blue sector summarizes mechanisms investigated in CIK oncology studies. The green sector summarizes findings from CIK or CIK-containing interventions, distinguishing antigen-loaded targets, infected or virus-transformed cells, and clinical observations. In the CMV peptide-loaded model, HLA class I, DNAM-1 and LFA-1 blockade reduced cytotoxicity, whereas NKG2D and NKp30 blockade did not inhibit antigen-specific killing. These findings should not be extrapolated to naturally infected targets without direct evaluation. The red sector separates infection-related findings in other lymphocytes from proposed applications requiring validation in CIK cells. Receptor expression is distinguished from functional involvement, and receptor symbols do not imply uniform expression across CIK preparations. Observations from combined cellular or antimicrobial treatments do not establish an independent CIK effect. Study-specific evidence and limitations are summarized in Table 2

In these contexts, the ability to sense and integrate molecular cues from the microenvironment is paramount. While innate immune cells and lymphocytes typically utilize germline-encoded pattern recognition receptors (PRRs) to detect conserved pathogen-associated molecular patterns (PAMPs) and danger-associated molecular patterns (DAMPs), thereby initiating the cytokine cascades that orchestrate cytotoxicity, CIK cells present a unique receptor profile that requires optimization. Within this framework, NKG2D and DNAM-1 recognize host-derived ligands, whereas TLR4 and TLR7 belong to the conventional PRR family. Although they consistently express DNAM-1 and NKG2D, other critical sensing receptors such as NKp30, NKp44 NKp46, NKG2C, and NKG2E often display low or variable expression. This phenotypic heterogeneity provides a rationale for investigating whether cytokine stimulation can modify receptor expression and function. Nevertheless, receptor expression alone does not establish functional involvement in antimicrobial activity. NKp30 contributes to CIK cytotoxicity against malignant targets, but its blockade did not inhibit CMV peptide-specific killing in the model examined [233]. Therefore, whether modulation of additional receptors improves infection-specific CIK activity remains to be determined. For instance, IL-15 stimulation increased TLR4 expression in CIK cells, and TLR4 knockdown impaired cytotoxicity against tumor cells, providing functional evidence in an oncology setting. Similarly, a TLR7 agonist enhanced the antitumor activity of CIK/NK preparations against K562 cells. These findings support investigation of TLR-dependent modulation in infectious settings, but neither study demonstrated that the corresponding receptor mediated CIK recognition or elimination of infected targets [233, 240–242, 245, 265–291]. Additionally, the DNAM-1 axis provides a crucial mechanism for pathogen recognition; its ligands, CD155 (PVR) and CD112 (Nectin-2), are markedly upregulated during infections including CMV, HIV, and *Echinococcus multilocularis*, while remaining absent on healthy tissues. DNAM-1 contributes to CIK cytotoxicity against malignant targets and to antigen-specific killing of CMV peptide-loaded autologous targets. The latter provides direct functional evidence in an infection-related antigen model, but does not establish DNAM-1-dependent recognition of naturally infected cells. Infection-related observations concerning DNAM-1 and its ligands in other experimental systems provide an additional rationale for investigation, rather than confirmation of the corresponding mechanism in CIK cells [233, 292–295]. In addition to these pathways, CD16-expressing CIK populations can mediate ADCC following engagement with tumor-targeting antibodies. This mechanism provides a rationale for investigating antibody-directed CIK activity against infected targets, with its contribution to target recognition and antimicrobial activity requiring direct evaluation in infection-specific models [230]. The experimental evidence supporting these recognition, activation, and effector pathways is summarized in Table 2 and illustrated in Fig. 3, distinguishing findings from infection related CIK studies, CIK oncology studies, and other cytotoxic lymphocytes, alongside proposed applications requiring direct evaluation. A recently discussed innovative approach involves expanding the classical CAR architecture by integrating accessory molecules that enhance pathogen detection and functional performance in infected environments. As a proposed strategy for infectious disease application, CAR constructs can be enriched with a variety of elements, including PRRs. These PRR-based or PRR-like CAR cells function in conjunction with additional cytokine and chemokine receptors modified co-receptors, and transcriptional regulators to improve cell trafficking, persistence, and effector functions within inflammatory microenvironments. These concept’s effects on pathogen recognition, target selectivity, and antimicrobial activity in CIK cells require direct experimental evaluation [296].

**Table 2:** Evidence supporting proposed recognition, activation, and effector pathways of CIK cells

Pathway	Evidence in CIK cells against tumors	CIK evidence in infection-related settings	Evidence from other lymphocytes	References
TCR	Antigen-specific T-cell function retained alongside non-MHC-restricted tumor cytotoxicity	CMV peptide-specific killing; Reduced killing following HLA class I blockade; Killing of CMV-infected fibroblasts by both CMV-directed and conventional preparations, so not attributable to antigen-specific recognition; following NKG2D blockade; Reduced killing of peptide-loaded targets following combined NKG2D and TCR blockade; EBV peptide-directed CIK activity against peptide-loaded targets; Cytotoxicity against autologous EBV-transformed lymphoblastoid cells, not exceeding that against an EBV-negative control target	MHC-restricted killing of virus-infected cells by conventional cytotoxic T lymphocytes;	[24, 233, 257, 258, 286]
NKG2D	Functional contribution to malignant-target killing; Reduced cytotoxicity following receptor blockade	High expression in virus-directed CIK cultures; No inhibition of CMV peptide-specific killing following NKG2D blockade; Contribution to other infection-related CIK responses unconfirmed	Direct fungal recognition and antifungal activity in NK cells	[185, 233, 236, 257, 258, 285]
DNAM-1	Contribution to selected malignant-target killing	Reduced CMV peptide-loaded target killing following DNAM-1 blockade; Contribution in an antigen-loaded model; Recognition of naturally infected cells through DNAM-1 unconfirmed	Direct binding to fungal Sap10; DNAM-1-dependent NK-cell activation	[233, 292, 294, 297]
NKp30	Functional contribution to selected malignant-target killing	No inhibition of CMV peptide-specific killing following NKp30 blockade; Infection-specific contribution otherwise unconfirmed	Direct fungal recognition and killing by NK cells	[184, 233, 298]
TLR4	Increased expression following IL-15 stimulation; Reduced tumor cytotoxicity following TLR4 knockdown	Infected-target recognition or killing not examined; Infection-specific role hypothetical	-	[288]
TLR7	Agonist-enhanced expansion and K562 cytotoxicity in mixed CIK/NK cultures	Infected-target recognition or killing not examined; CIK-specific infection role hypothetical	NK cells included in mixed cultures; Independent NK-cell contribution unresolved	[290]
CD16 mediated ADCC	Variable CD16 expression; Antibody-dependent killing of malignant targets	ADCC against infected targets unconfirmed; Proposed application requiring direct validation	NK-cell ADCC against *P. falciparum*-infected erythrocytes	[214, 230]
CD56	-	Infection-specific function unconfirmed	Engagement with hyphal cell-wall polysaccharide galactosaminogalactan (GAG), leading to NK cell activation.	[187]
Cytotoxic secretion	Granule-mediated tumor cytotoxicity	*P. falciparum* suppression retained in transwell cultures; Soluble-effector contribution supported; Recognition receptor and infected-erythrocyte selectivity unresolved	Cytotoxic effector activity against *Aspergillus fumigatus* in engineered CAR T cells; GAG-induced NK-cell degranulation and cytotoxic effector secretion through CD56 Cytotoxic effector activity of Vγ9Vδ2 T cells against P. falciparum-infected erythrocytes	[58, 122, 187, 228, 233, 241, 275]

Evidence refers to the experimental systems examined in the cited studies. Receptor expression alone does not establish functional involvement. Findings in other lymphocytes do not demonstrate the corresponding mechanism in CIK cells

In summary, CIK cells represent a candidate approach for infectious disease immunotherapy, supported by preliminary findings in selected infections. By combining the proliferative capacity of T cells with the broad, stress-ligand-mediated recognition of NK cells, they offer a rationale for investigating their activity involving immune evasion. Clinical tolerability and manufacturing feasibility have been evaluated in selected CIK studies, predominantly in oncology. These observations, together with evidence of retained function after cryopreservation, support further investigation but do not establish superior safety, feasibility, cost-effectiveness or accessibility in infectious diseases.

Despite their promise, critical challenges remain. Modulation of activating receptors and PRRs may influence CIK cell responses and their versatility in pathogen detection, but whether increased expression improves recognition and control of a particular infection remains to be established. Future studies should determine which recognition pathways contribute to antimicrobial activity and whether their modulation improves target specificity and pathogen control. Crucially, the investigation of CIK cells must expand beyond oncology into the realm of infectious diseases. Further infection-specific studies are required to determine whether the biological properties of CIK cells translate into selective antimicrobial activity and clinical benefit and to establish their comparative efficacy and safety in the intended patient populations.

## Data Availability

No datasets were generated or analysed during the current study.
